# Macrophage SR-BI mediates efferocytosis via Src/PI3K/Rac1 signaling and reduces atherosclerotic lesion necrosis[S]

**DOI:** 10.1194/jlr.M056689

**Published:** 2015-08

**Authors:** Huan Tao, Patricia G. Yancey, Vladimir R. Babaev, John L. Blakemore, Youmin Zhang, Lei Ding, Sergio Fazio, MacRae F. Linton

**Affiliations:** * Department of Medicine, Atherosclerosis Research Unit, Division of Cardiovascular Medicine, Vanderbilt University School of Medicine, Nashville, TN 37232; § Department of Pharmacology, Vanderbilt University School of Medicine, Nashville, TN 37232; † Department of Medicine, Physiology, and Pharmacology, Center of Preventive Cardiology, Oregon Health and Science University, Portland, OR 97239

**Keywords:** atherosclerosis, apoptosis, inflammation, scavenger receptor class B type I, Src, phosphoinositide 3-kinase, Ras-related C3 botulinum toxin substrate 1

## Abstract

Macrophage apoptosis and efferocytosis are key determinants of atherosclerotic plaque inflammation and necrosis. Bone marrow transplantation studies in ApoE- and LDLR-deficient mice revealed that hematopoietic scavenger receptor class B type I (SR-BI) deficiency results in severely defective efferocytosis in mouse atherosclerotic lesions, resulting in a 17-fold higher ratio of free to macrophage-associated dead cells in lesions containing SR-BI^−/−^ cells, 5-fold more necrosis, 65.2% less lesional collagen content, nearly 7-fold higher dead cell accumulation, and 2-fold larger lesion area. Hematopoietic SR-BI deletion elicited a maladaptive inflammatory response [higher interleukin (IL)-1β, IL-6, and TNF-α lower IL-10 and transforming growth factor β]. Efferocytosis of apoptotic thymocytes was reduced by 64% in SR-BI^−/−^ versus WT macrophages, both in vitro and in vivo. In response to apoptotic cells, macrophage SR-BI bound with phosphatidylserine and induced Src phosphorylation and cell membrane recruitment, which led to downstream activation of phosphoinositide 3-kinase (PI3K) and Ras-related C3 botulinum toxin substrate 1 (Rac1) for engulfment and clearance of apoptotic cells, as inhibition of Src decreased PI3K, Rac1-GTP, and efferocytosis in WT cells. Pharmacological inhibition of Rac1 reduced macrophage efferocytosis in a SR-BI-dependent fashion, and activation of Rac1 corrected the defective efferocytosis in SR-BI^−/−^ macrophages. Thus, deficiency of macrophage SR-BI promotes defective efferocytosis signaling via the Src/PI3K/Rac1 pathway, resulting in increased plaque size, necrosis, and inflammation.

Scavenger receptor class B type I (SR-BI) is an HDL receptor that promotes bidirectional flux of free cholesterol between cells and HDL, as well as selective uptake of HDL cholesteryl ester into hepatocytes and steroidogenic cells (1, 2). Single deletion of mouse SR-BI accelerates atherosclerosis, whereas combined deletion of SR-BI and ApoE mimics several features of human coronary disease, including occlusive atherosclerosis, myocardial infarction, and premature death (3). These anti-atherosclerotic effects are only in part due to hepatic SR-BI regulation of HDL cholesterol metabolism (4), with mounting evidence supporting independent atheroprotective functions of macrophage SR-BI. Transplantation of SR-BI^−/−^ bone marrow (BM) into LDLR^−/−^ mice accelerates atherosclerosis development (5). Similarly, transplantation of SR-BI^−/−^/ApoE^−/−^ BM into ApoE^−/−^ mice enhances atherosclerotic lesion formation (6).

The mechanisms by which macrophage SR-BI influences atherosclerosis have not been elucidated. Macrophage cell death and the efferocytosis of apoptotic cells are important determinants of inflammation and atherosclerotic lesion development (7). Intimal macrophages undergo apoptosis due to lipid toxicity, oxidative stress, and signaling from bioactive molecules. Failure of apo­ptotic cell clearance promotes secondary necrosis, with release of proteolytic enzymes and other active molecules into the surrounding tissue causing inflammation and plaque destabilization (7, 8). Interestingly, SR-BI binds phosphatidylserine (PS) and oxidized phospholipids; both serve as “eat me” signals on apoptotic cells (9). However, macrophages have a variety of receptors to recognize different apoptotic cells and ligands for phagocytosis (7), and whether macrophage SR-BI mediates efferocytosis in the setting of atherosclerosis remains unknown.

In this study, we examined the hypothesis that macrophage SR-BI deficiency impairs efferocytosis of apoptotic cells in atherosclerotic lesions, promoting lesion inflammation and necrosis. To accomplish this, we performed BM transplantation studies using both ApoE^−/−^ and LDLR^−/−^ recipient mice. Our in vitro studies show that macrophage SR-BI functions as a PS receptor for recognition and clearance of apoptotic cells. Importantly, hematopoietic SR-BI deletion caused marked accumulation of noninternalized apoptotic cells in atherosclerotic lesions, resulting in increased necrosis. In addition, hematopoietic SR-BI deletion increased serum inflammatory cytokine levels and enhanced expression of proinflammatory genes in macrophage foam cells. Finally, we determined that macrophage SR-BI mediates efferocytosis via a novel proto-oncogene tyrosine-protein kinase (Src)/phosphoinositide 3-kinase (PI3K)/Ras-related C3 botulinum toxin substrate 1 (Rac1) signaling pathway. Taken together, our results show that macrophage SR-BI protects against atherosclerotic lesion necrosis and progression by regulating efferocyte signaling and macrophage survival.

## MATERIALS AND METHODS

A detailed description of all experimental methods is available online in the supplementary data.

### Mice

SR-BI^+/−^ (1:1 mixed C57BL/6×S129 genetic background) were obtained from the Jackson Laboratory and backcrossed over 10 generations onto the C57BL/6 background. Animal protocols were performed according to the regulations of Vanderbilt University’s Institutional Animal Care and Usage Committee. Mice were maintained on chow or a Western-type diet containing 21% milk fat and 0.15% cholesterol (Teklad).

### Atherosclerosis analyses

Female ApoE^−/−^ or LDLR^−/−^ mice were lethally irradiated and transplanted with 5 × 10^6^ BM cells from SR-BI^+/+^ApoE^−/−^ and SR-BI^−/−^ApoE^−/−^ (DKO) mice (10) or BM from WT, SR-BI^−/−^, ApoE^−/−^, and DKO mice. After 4 weeks, the mice were placed on a Western-type diet for 8 weeks or 16 weeks. The extent of atherosclerosis was examined both in Oil-Red-O-stained cross-sections of the proximal aorta and by en face analysis using the KS300 imaging system (Kontron Elektronik GmbH) (10).

### Serum lipids, lipoprotein profiles, and cytokines

Mice were fasted for 4 h, and total serum cholesterol and triglycerides were determined by enzymatic methods (Raichem). Serum levels of interleukin (IL)-1β, IL-6, IL-10, transforming growth factor β (TGF-β), and TNF-α were determined by ELISA (eBioscience or R&D Systems).

### Cell culture and transfections

Peritoneal macrophages were isolated (6) from mice reconstituted with WT, SR-BI^−/−^, ApoE^−/−^, and DKO BM. Mouse thymocytes were isolated as described (11). For transient transfections, Lipofectamine LTX and Plus reagent (Invitrogen) with pCMV6-mSR-BI or scramble pCMV6 plasmid (OriGene Technologies) were applied to macrophages. For the gene knockdown assay, WT and SR-BI^−/−^ peritoneal macrophages were infected with short hairpin high-mobility group protein B1 (shHMGB1) or scramble shRNA lentivirus (Origene Technologies) using the manufacturer’s protocol.

### Analyses of efferocytosis and membrane ruffling

For efferocytosis assays, thymocytes were labeled with carboxyfluorescein diacetate, succinimidyl ester (CFDA SE) green cell tracer (Invitrogen) and apoptosis was induced with 600 rad irradiation. Apoptotic cells were added onto cultured phagocytes in vitro or injected into mice in vivo for 2 h. The phagocytes were stained with CMTPX red cell tracer (Invitrogen). The efferocytosis of apoptotic cells was then visualized using fluorescence microscopy and quantitated by flow cytometry (12, 13). For anal­ysis of membrane ruffling, phagocytes were incubated for 20 min with or without apoptotic thymocytes. After washing away apo­ptotic thymocytes, the cells were then fixed, permeabilized, stained with rhodamine phalloidin (Cytoskeleton, Inc.), and counterstained with Hoechst.

### Analyses of lesion necrosis, cell death, and efferocytosis

Necrosis was quantitated by hematoxylin and eosin (H and E) stain. For analysis of cell death and efferocytosis, proximal aortic cryosections were dual stained with terminal deoxyribonucleotidyl transferase-mediated dUTP nick-end labeling (TUNEL) and macrophage antibody; nuclei were counterstained with Hoechst. The free versus macrophage-associated TUNEL stain in the sections were quantitated and normalized to total lesion area as described (13).

### Phospholipid-SR-BI binding assay

PS (Avanti) was loaded onto nitrocellulose membrane-C (HyBond) (14) and incubated with glutathione S-transferase (GST)-tagged SR-BI (Novus) or GST alone. The bound protein was detected by anti-SR-BI (Novus) or anti-GST (Novus) and secondary antibody (Sigma). For a cell-based assay, apoptosis was induced in SR-BI^−/−^ macrophages, and exposure of PS was analyzed by annexin V directly or by incubation with GST-SR-BI protein or GST alone. SR-BI was detected using anti-SR-BI antibody.

### Immunoprecipitation, Western blotting, and Rac1/RhoA-GTP activity assay

Macrophage lysates were incubated with antibody against either SR-BI or Src, and then with magnetic beads (Invitrogen). The magnetic beads were boiled with sample buffer at 70°C for 5 min, and the supernatant was used for detecting Src or SR-BI by immunoblotting. For Western blot, primary antibodies specific for SR-BI (Novus), total Akt, pAkt, PI3K p85, HMGB1, Src, pSrc, Na^+^/K^+^-ATPase (Cell Signaling), Rac1 (Upstate), RhoA (Cytoskeleton), Lamp-1 (Millipore), and β-actin (Sigma) were applied. To assess activation of Rac1/RhoA, aliquots of the cell extracts were loaded with GTPγS, immunoprecipitated with PAK1/Rhotekin-PBD-agarose at 4°C for 60 min, and immunoblotted for Rac1/RhoA following the manufacturer’s protocol (Millipore/Cytoskeleton).

### Plasma membrane protein and phagosome preparation

Plasma membrane proteins were extracted using a plasma membrane protein extraction kit (BioVision). Phagosomes were isolated as described (15) using latex beads (Sigma).

### RNA isolation and real-time RT-PCR

Total RNA was purified using Aurum Total RNA kit (Bio-Rad). cDNA was synthesized with reverse transcriptase (Bio-Rad). Relative quantitation of target mRNA was performed using specific primers (supplementary Table), SYBR probe (Bio-Rad), and iTaqDNA polymerase (Bio-Rad) on an IQ5 thermocylcer (Bio-Rad) and normalized to β-actin.

### Statistical analysis

Data are presented as mean ± SEM. Differences between mean values were determined by Kruskal-Wallis test (Bunn’s multiple comparison), one-way ANOVA (Bonferroni’s post test), Mann-Whitney test, and Student’s *t*-test using GraphPad PRISM.

## RESULTS

### Deletion of macrophage SR-BI impairs efferocytosis in vitro and in vivo

**Fig. 1.**
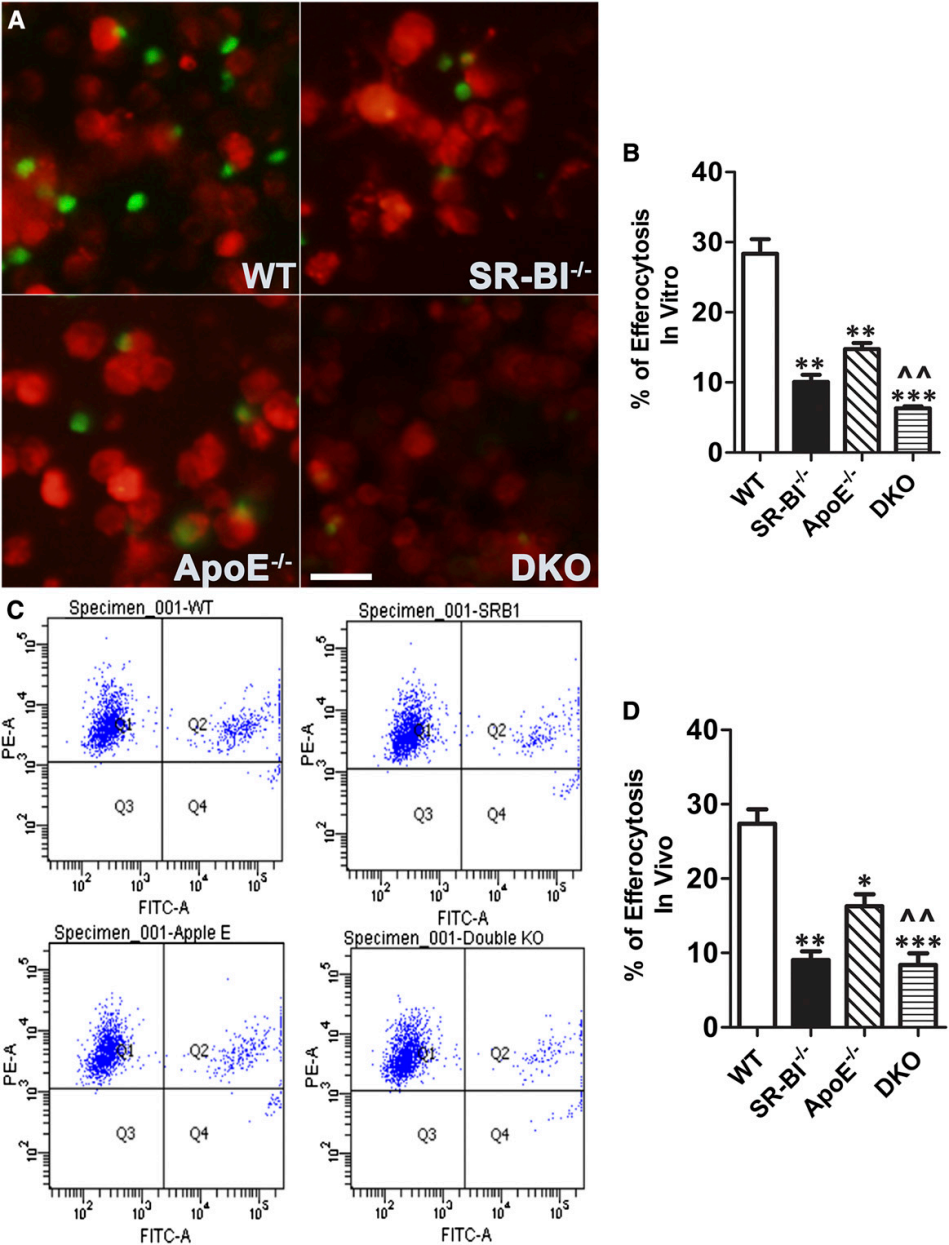
Deletion of macrophage SR-BI impairs efferocytosis in vitro and in vivo. A, B: CFDA-SE-labeled apoptotic WT thymocytes (green) were added onto CMPTX-labeled WT, SR-BI^−/−^, ApoE^−/−^, and DKO macrophages (red) at a ratio of 10:1 and incubated for 2 h. After vigorous washing with PBS, efferocytosis of apoptotic cells was visualized and quantitated using fluorescence microscopy and flow cytometry. The data are the mean ± SEM for three experiments. **P < 0.01, ***P < 0.001 compared with WT, ^^*P* < 0.01 compared with either SR-BI^−/−^ or ApoE^−/−^. Scale bar = 50 μm. C: CFDA-SE-labeled apoptotic WT thymocytes (1 × 10^9^ cells) were injected intraperitoneally into LDLR^−/−^ mice reconstituted with WT, SR-BI^−/−^, ApoE^−/−^, and DKO BM that had been injected 2 days previously with 3 ml of 3.0% thioglycollate. Two hours later, peritoneal cells were plated onto wells and stained with CMPTX red cell tracer. After adherence, the macrophages were scraped and efferocytosis of apoptotic cells was quantitated using flow cytometry. The data are the mean ± SEM (n = 9 per group). * P < 0.05, **P < 0.01, ***P < 0.001 compared with WT, ^^*P* < 0.05 DKO compared with ApoE^−/−^.

To determine the role of macrophage SR-BI in phagocytosis of apoptotic cells, we examined the in vitro phagocytic engulfment of CFDA-SE-labeled apoptotic thymocytes by WT, ApoE^−/−^, SR-BI^−/−^, and DKO peritoneal macrophages (**Fig. 1A, B**). Compared with WT macrophages, the efferocytosis of apoptotic cells was reduced by 64.4% (*P* < 0.01), 47.9% (*P* < 0.01), and 77.7% (*P* < 0.001) in SR-BI^−/−^, ApoE^−/−^, and DKO macrophages, respectively. The impairment in efferocytosis was not due to enhanced apoptosis induced by the thymocytes because, after incubation with the thymocytes, less than 0.5% of the cells in all cultures were positive for annexin V (data not shown). In addition, in vivo examination of the phagocytosis of CFDA-SE-labeled apoptotic thymocytes by macrophages in the peritoneal cavity of LDLR^−/−^ mice reconstituted with WT, SR-BI^−/−^, ApoE^−/−^, and DKO BM (Fig. 1C, D) showed that efferocytosis of apoptotic cells was reduced by 47.4% (*P* < 0.01), 29.1% (*P* < 0.05), and 66.7% (*P* < 0.001) in SR-BI^−/−^, ApoE^−/−^, and DKO macrophages, respectively. These results demonstrate that macrophage SR-BI plays a critical role in efferocytosis of apoptotic cells.

### Deletion of macrophage SR-BI enhances inflammation

**Fig. 2.**
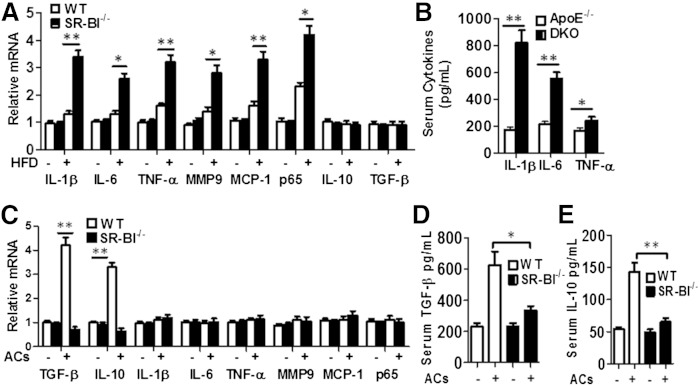
Hematopoietic SR-BI deletion results in a maladaptive inflammatory response. A: Pro- and anti-inflammatory gene expression was measured by real-time RT-PCR in macrophages isolated from LDLR^−/−^ mice transplanted with WT or SR-BI^−/−^ BM. Recipient mice were fed chow or high fat diet (HFD) for 16 weeks. The data are presented as mean ± SEM (n = 6 per group) with statistical difference between WT and SR-BI^−/−^ levels indicated by *P < 0.05, **P < 0.01. B: ApoE^−/−^ and ApoE^−/−^SR-BI^−/−^ (DKO) BM recipient ApoE^−/−^ mice were fed a high fat diet for 8 weeks, and then serum IL-1β, IL-6, and TNF-α levels were measured by ELISA. Data are presented as mean ± SEM (n = 6 per group). *P < 0.05, ** *P* < 0.01. C: The mRNA levels of pro- and anti-inflammatory genes were analyzed by real-time RT-PCR in macrophages after incubation with or without apoptotic cells (ACs). The data are presented as mean ± SEM of three experiments. **P < 0.01. D, E: The serum levels of IL-10 and TGF-β were measured by ELISA before and 2 h after ip injection of apoptotic thymocytes in LDLR^−/−^ mice reconstituted with WT and SR-BI^−/−^ hematopoietic cells on a normal chow diet. Data are presented as mean ± SEM (n = 6 per group). *P < 0.05, ** *P* < 0.01.

As defective phagocytosis of apoptotic cells results in secondary cellular necrosis and maladaptive inflammation, we next examined the effects of SR-BI deficiency on inflammation in vitro and in vivo. Peritoneal macrophages were isolated from LDLR^−/−^ mice transplanted with either WT or SR-BI^−/−^ BM and fed either a chow or Western-type diet for 16 weeks (**Fig. 2**). Both WT and SR-BI^−/−^ macrophages from mice on the Western diet had higher mRNA levels of IL-1β, IL-6, TNF-α, matrix metaolloproteinase 9 (MMP-9), monocyte chemotactic protein 1 (MCP-1), and p65 nuclear factor (NF)-κB (Fig. 2A, *P* < 0.05 or 0.01) compared with cells from mice on the chow diet. However, SR-BI^−/−^ macrophages had dramatically increased expression of all proinflammatory genes compared with WT cells from mice on the atherogenic diet. Compared with mice receiving ApoE^−/−^ BM, DKO BM recipient mice had significantly higher serum levels of IL-1β, IL-6, and TNF-α (Fig. 2B). After incubation with apoptotic cells, SR-BI^−/−^ macrophages showed defective efferocytosis (Fig. 1) and significantly decreased mRNA expression of the anti-inflammatory cytokines, IL-10 and TGF-β (Fig. 2C; *P* < 0.01); similarly, serum levels of IL-10 and TGF-β were reduced in response to peritoneal injection of apoptotic thymocytes in LDLR^−/−^ mice reconstituted with SR-BI^−/−^ versus WT hematopoietic cells (Fig. 2D, E). Taken together, these studies show that macrophage SR-BI de­ficiency elicits a maladaptive inflammatory response in vitro and also in vivo in two different murine models of atherosclerosis.

### Hematopoietic SR-BI deficiency promotes increased atherosclerotic lesion development and cell death

**Fig. 3.**
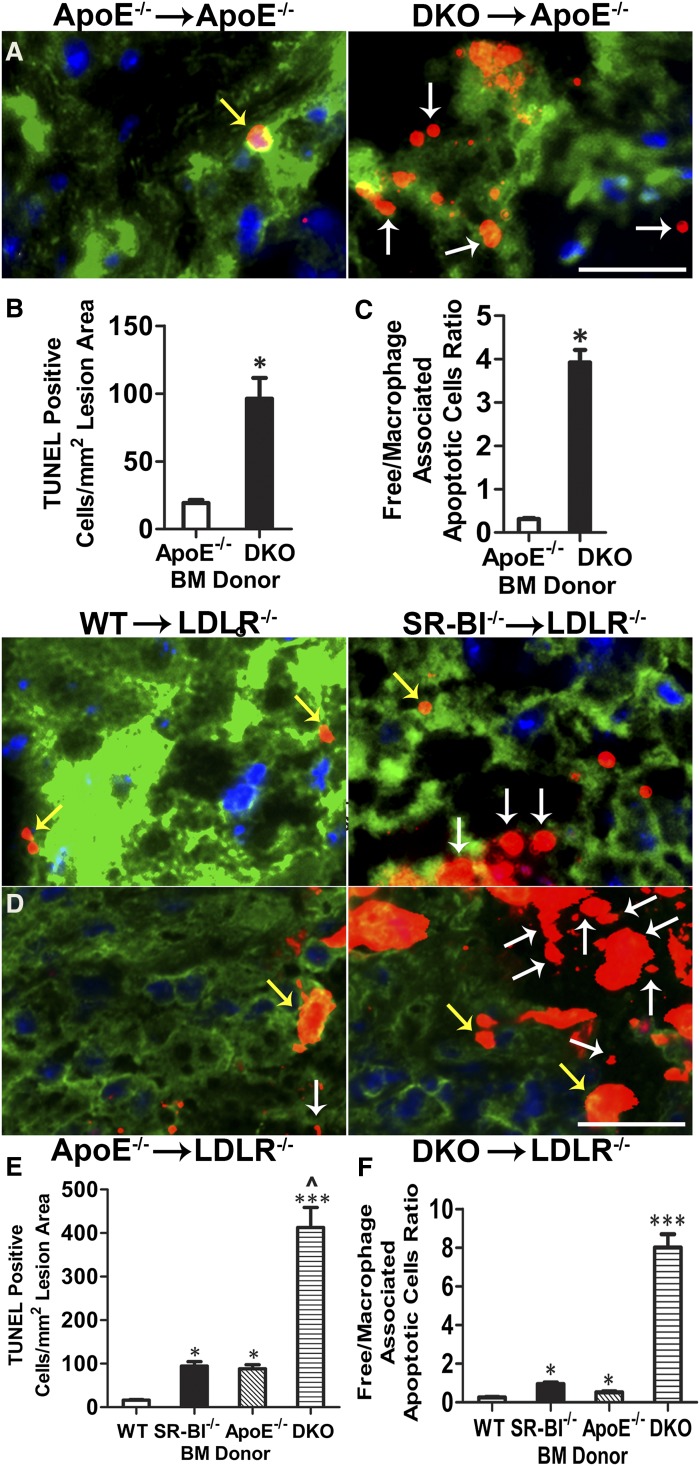
Hematopoietic SR-BI deficiency promotes atherosclerotic lesion cell death and impairs efferocytosis in ApoE^−/−^ and LDLR^−/−^ BM transplant-recipient mice. Dead cells were detected by TUNEL staining of proximal aorta sections from ApoE^−/−^ mice reconstituted with ApoE^−/−^ or DKO BM and fed a Western diet for 8 weeks, and from LDLR^−/−^ mice reconstituted with WT, SR-BI^−/−^, ApoE ^−/−^, or DKO BM and fed a Western diet for 16 weeks. Micrographs show merged images of nuclei (Hoechst, blue), TUNEL staining (red), and macrophage cytoplasm (green) in aortic root sections from recipient ApoE^−/−^ (A) or LDLR^−/−^ (D) mice. Yellow arrows represent macrophage-associated TUNEL stain and white arrows represent free TUNEL stain. Scale bar = 50 μm. Quantitation of the number of TUNEL-positive nuclei in proximal aorta sections from recipient ApoE^−/−^ mice (B) or LDLR^−/−^ mice (C). Data are indicated as mean ± SEM (n = 8 per group). *P < 0.05. Efferocytosis was examined by quantitating the free versus macrophage-associated TUNEL-positive cells in proximal aorta sections of recipient ApoE^−/−^ (E) or LDLR^−/−^ mice (F) (n = 8 per group). *P < 0.05, ***P < 0.001.

To evaluate the role of macrophage SR-BI in atherosclerotic lesion development and cell death, 8-week-old ApoE^−/−^ mice were transplanted with either ApoE^−/−^ or DKO BM and fed a Western diet for 8 weeks. Similar to our previous studies (6), ApoE^−/−^ mice receiving DKO BM had 2.7-fold more lesion area in the proximal aorta compared with mice receiving ApoE^−/−^ BM (221.83 ± 30.33 × 10^3^ μm^2^ versus 81.83 ± 11.64 × 10^3^ μm^2^, *P* < 0.001, supplementary Fig. 1A, B). In addition, en face evaluation of pinned-out aortas showed 56% more atherosclerosis in DKO→ApoE^−/−^ mice versus ApoE^−/−^→ApoE^−/−^ mice (*P* < 0.001, supplementary Fig. 1C, D). Lesion cell death was examined by staining with tetramethylrhodamine (TMR) red TUNEL, counterstaining DNA with Hoechst (supplementary Fig. 2, **Fig. 3A**), and macrophages using a rabbit anti-macrophage antibody. Quantitation of the TUNEL stain showed that lesions of DKO→ApoE^−/−^ mice contained 6.7-fold more dead cells per square millimeter of lesion area compared with the lesions of ApoE^−/−^→ApoE^−/−^ mice (*P* < 0.05, Fig. 3B). The TUNEL stain was localized to macrophage-enriched regions in both groups of mice (supplementary Fig. 2). However, the lesions of DKO→ApoE^−/−^ mice had significant TUNEL stain localized in regions where Hoechst staining of nuclei was fragmented, diffuse, and/or faded (supplementary Fig. 2). To examine the impact of macrophage deficiency of SR-BI in the presence or absence of macrophage ApoE expression on atherosclerotic lesion development and cell death (5, 6, 10), 8-week-old LDLR^−/−^ mice were transplanted with BM from WT, SR-BI^−/−^, ApoE^−/−^, and DKO mice and fed a Western diet for 16 weeks. Compared with LDLR^−/−^ mice receiving WT BM (supplementary Fig. 3), lipid-rich lesion area in the proximal aorta was increased by 156% (*P* < 0.05), 150% (*P* < 0.05), and 403% (*P* < 0.001) in mice receiving SR-BI^−/−^, ApoE^−/−^, and DKO BM, respectively (supplementary Fig. 3A, B). Similarly, compared with LDLR^−/−^ mice receiving WT BM (supplementary Fig. 3D, E), the lesion area in whole aortas was increased by 231% (*P* < 0.05), 214% (*P* < 0.05), and 431% (*P* < 0.001) in LDLR^−/−^ mice receiving SR-BI^−/−^, ApoE^−/−^, and DKO BM, respectively. The lesions of SR-BI^−/−^, ApoE^−/−^, or DKO→LDLR^−/−^ mice contained 4.6-, 3.5-, and 24-fold more TUNEL stain compared with lesions of WT→LDLR^−/−^ mice (supplementary Fig. 4; Fig. 3D, E). In lesions of all four mouse groups, TUNEL stain was localized to macrophage-enriched regions (Fig. 3D), but the lesions of DKO→LDLR^−/−^ mice also had a large amount of TUNEL stain in areas where Hoechst staining of nuclei appeared diffuse, heavily fragmented, and/or faded (supplementary Fig. 4). Consistent with the increased numbers of dead cells, the live macrophage content was significantly decreased in SR-BI^−/−^ and DKO lesions versus WT and ApoE^−/−^ lesions (supplementary Fig. 3C). For both ApoE^−/−^ and LDLR^−/−^ recipient mice, plasma lipid and lipoprotein levels were similar among the recipient groups (data not shown).

### Effects of hematopoietic SR-BI deletion on lesional efferocytosis, apoptosis, necrosis, collagen, and fibrous cap thickness

**Fig. 4.**
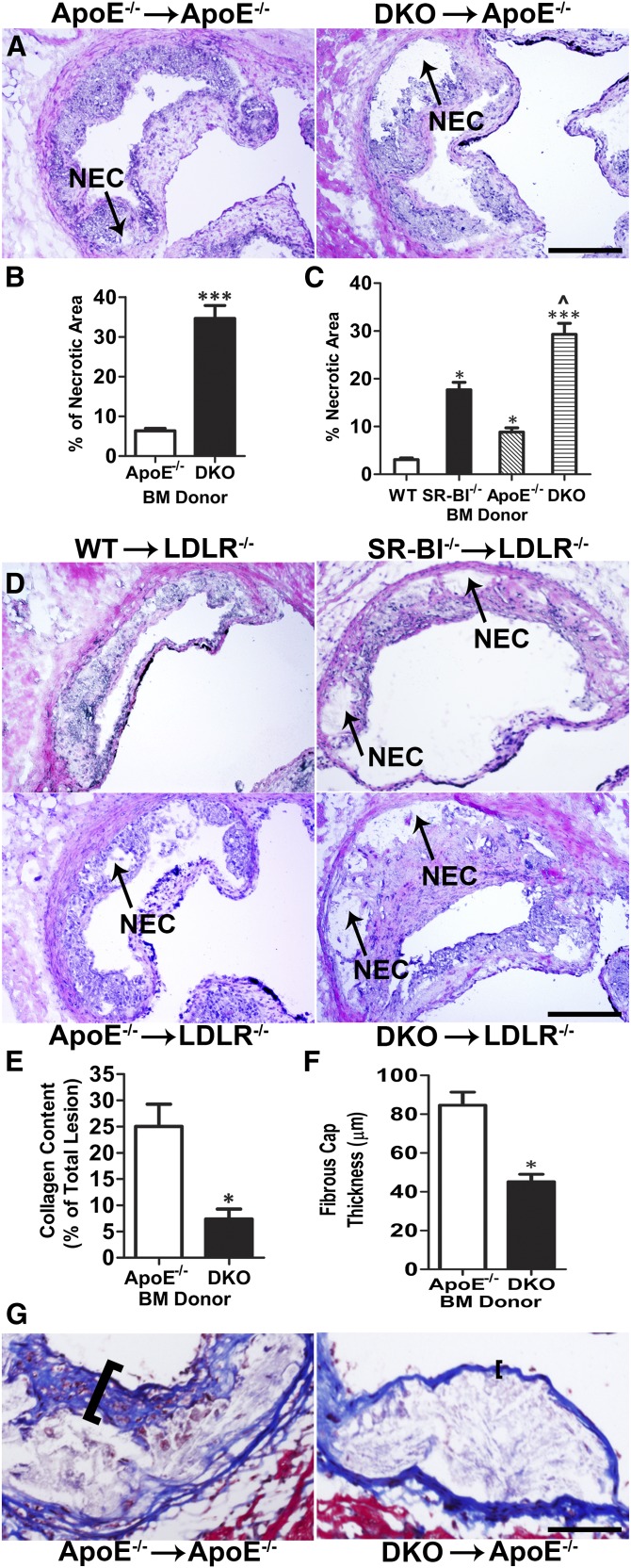
Macrophage SR-BI deficiency increases necrosis in atherosclerotic lesions of ApoE^−/−^ and LDLR^−/−^ mice. Necrosis was analyzed by H and E staining. Representative images of H and E staining of proximal aorta sections from ApoE^−/−^ or DKO BM-recipient ApoE^−/−^ mice (A) and from WT, SR-BI^−/−^, ApoE^−/−^, or DKO BM-recipient LDLR^−/−^ mice (D). Arrows denote necrotic (NEC) area. The percent necrotic area was quantitated using ImageJ software (n = 12 per group). ***P < 0.001 (B) and *P < 0.05 and ***P < 0.001 compared with WT, respectively, ^*P* < 0.01 DKO compared with ApoE^−/−^ (C). The collagen content (E) and fibrous cap thickness (F) were analyzed by Masson’s trichrome stain and quantitated using ImageJ software in cross-section from above ApoE^−/−^ mice and representative images are shown in (G) (n = 8 per group). *P < 0.05, scale bar = 100 μm.

We next examined whether the increased lesional accumulation of dead cells resulting from deletion of SR-BI was due to defective efferocytosis. Staining of lesions for apoptosis, nuclei, and macrophages (Fig. 3A–F) enabled the quantitation of free versus macrophage-associated TUNEL stain, as previously described (13). The ratio of free to macrophage-associated dead cells was 17-fold higher in lesions of DKO→ApoE^−/−^ mice compared with ApoE^−/−^→ApoE^−/−^ mice (Fig. 3C). Consistent with defective lesional efferocytosis, we found that significant TUNEL stain was localized to regions poorly stained with Hoechst (supplementary Fig. 2) and the percent necrotic area was 5.2-fold (*P* < 0.01) greater in lesions of DKO→ApoE^−/−^ mice (**Fig. 4A, B**). Deletion of macrophage SR-BI also caused defective efferocytosis in lesions of LDLR^−/−^ recipient mice, where SR-BI^−/−^, ApoE^−/−^, or DKO→LDLR^−/−^ mice contained 4.4-, 2.3-, and 37-fold more free to macrophage-associated TUNEL stain compared with lesions of WT→LDLR^−/−^ mice (Fig. 3D, F), leading to dramatic increases in necrotic area (Fig. 4C, D). The necrotic area was increased by 5.7-, 2.8-, and 9.5-fold in LDLR^−/−^ mice reconstituted with SR-BI^−/−^, ApoE^−/−^, or DKO BM compared with WT→LDLR^−/−^ mice. As susceptibility to apoptosis could contribute to increased TUNEL-positive cells and necrosis observed in lesions of recipients of SR-BI^−/−^ BM, we also determined the levels of macrophage active caspase 3 in lesions of LDLR^−/−^ recipient mice (supplementary Fig. 5). The levels of macrophage active caspase 3 were increased by 2.4- (*P* < 0.01), 2.0- (*P* < 0.05), and 3.1-fold (*P* < 0.01) in lesions of LDLR^−/−^ mice transplanted with SR-BI^−/−^, ApoE^−/−^, or DKO BM compared with plaque containing WT cells, suggesting that both ApoE and SR-BI impact lesion macrophage apoptosis susceptibility. Interestingly, the lesions containing DKO cells only had 1.5-fold more active caspase 3 compared with ApoE^−/−^ lesions (supplementary Fig. 5). Thus, the impact of SR-BI and ApoE deficiency on apoptosis susceptibility likely contributes to the increased lesional TUNEL-positive cells and necrosis. However, compared with the effects on lesion active caspase 3 levels, the accumulation of free TUNEL-positive cells (Fig. 3F**)** was more pronounced in SR-BI^−/−^ versus WT lesions (4.3-fold) and DKO versus ApoE^−/−^ lesions (16.2-fold), indicating that defective efferocytosis is a major contributor to lesion progression and necrosis in SR-BI-deficient lesions. One key feature of plaque instability is collagen and fibrous cap reduction or depletion. Dying macrophages are a major source of proteases that originate from the necrotic core and degrade the extracellular matrix and induce plaque rupture. Therefore, we analyzed the collagen content and fibrous cap thickness in atherosclerotic lesions by Masson’s trichrome stain. We observed that hematopoietic SR-BI deficiency caused 65.2 and 48.5% reductions in lesion collagen content and fibrous cap thickness in DKO→ApoE^−/−^ mice compared with ApoE^−/−^→ApoE^−/−^ mice (Fig. 4E–G), suggesting that hematopoietic SR-BI deficiency contributes to unstable plaque formation.

### SR-BI protein interacts with PS and localizes in phagosomes

The observation that the combined deletion of macrophage SR-BI and ApoE impairs efferocytosis beyond the single deletion of either gene suggests that SR-BI mediates efferocytosis through pathways that are both dependent and independent of interaction with ApoE. Therefore, we examined the possibility that macrophage SR-BI interacts with apoptotic cell PS to initiate efferocytosis. We first compared the binding of GST-tagged SR-BI to PS in nonapoptotic control or apoptotic SR-BI^−/−^ macrophages (supplementary Fig. 6A). Both annexin V- and GST-tagged SR-BI bound to the surface of apoptotic SR-BI^−/−^ macrophages and not on nonapoptotic cells, suggesting that SR-BI interacts with membrane PS of apoptotic cells. Using a direct phospholipid SR-BI binding assay, we confirmed that SR-BI is a PS receptor. In contrast to GST protein, GST-tagged SR-BI interacted with nitrocellulose membrane PS in a dose-dependent fashion (supplementary Fig. 6B). The interaction with PS was specific, as GST-tagged SR-BI interacted poorly with other phospholipids, including phosphatidylcholine, phosphatidylglycerol, sphingomyelin, and phosphatidylethanolamine (data not shown). Together, these data indicate that SR-BI functions directly as a receptor for apoptotic cells by interacting with apoptotic cell PS. Consistent with SR-BI directly mediating the phagocytosis of apoptotic cells, SR-BI protein was enriched in phagosomes (supplementary Fig. 6C) of WT macrophages exposed to latex beads.

### Macrophage SR-BI regulates efferocytosis via Src/PI3K/Rac1 signaling

**Fig. 5.**
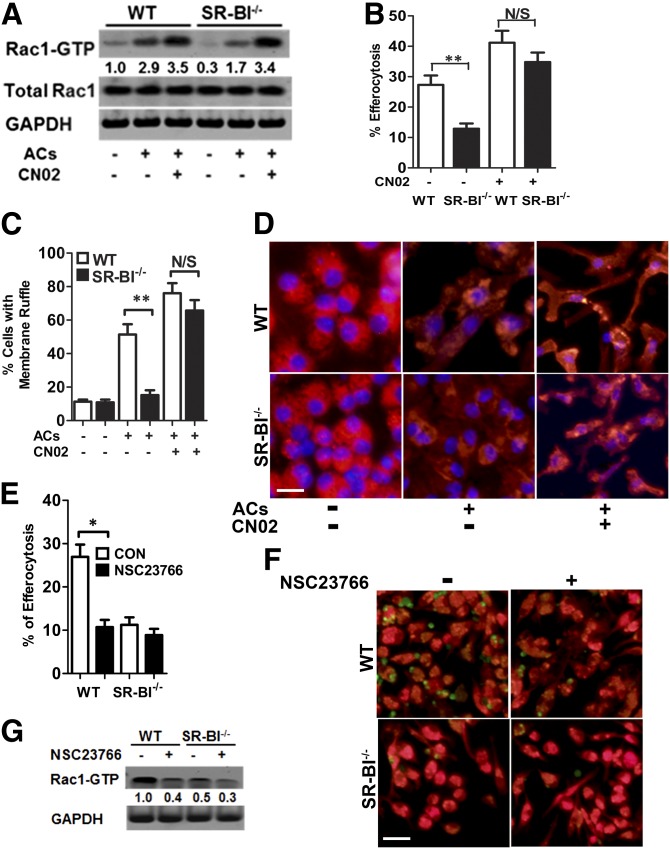
Effects of Rac1 activation on efferocytosis and membrane ruffling in WT and SR-BI^−/−^ macrophages. A: Immunoblotting analysis of Rac1-GTP, total Rac1, and GAPDH in WT or SR-BI^−/−^ macrophages treated with or without apoptotic cells (ACs) and/or CN02 (Rac1 activator, 1 unit). B: Efferocytosis was measured as above in WT and SR-BI^−/−^ macrophages in the presence or absence of CN02. C: Quantitation of cells with membrane ruffling in WT and SR-BI^−/−^ macrophages treated with or without ACs and/or CN02 as described in the Materials and Methods. D: Representative images of cells with membrane ruffling in WT and SR-BI^−/−^ macrophages treated as above. Cell F-actin was stained with rhodamine-labeled phalloidin and visualized under fluorescence microscopy. E, F: Efferocytosis was measured by flow cytometry in WT and SR-BI^−/−^ macrophages in the presence or absence of the Rac1 inhibitor, NSC23766. F: Images show phagocytes (red) and apoptotic cells (green). The data are presented as mean ± SEM of three experiments (B, C, E). G: Rac1 activity was detected by immunoblot analysis. A, G: Data are representative and numbers are the mean of three experiments. *P < 0.05, **P < 0.01, scale bar = 50 μm.

Efferocytosis is precisely regulated in prokaryotic and eukaryotic cells with evolutionarily overlapping signaling pathways leading to Rac1 activation and cytoskeletal rearrangements for engulfment. Therefore, we examined Rac1 activation in WT and SR-BI^−/−^ macrophages and the effects of pharmacological activation of Rac1 on membrane ruffling and efferocytosis (**Fig. 5**). Upon incubation with apoptotic cells, activation of Rac1, detected as Rac1-GTP, was markedly increased in WT cells compared with SR-BI^−/−^ cells (Fig. 5A). Consistent with defective efferocytosis and decreased Rac1-GTP in SR-BI^−/−^ macrophages, incubation of SR-BI^−/−^ macrophages with apoptotic thymocytes did not increase the number of cells with ruffled membranes (Fig. 5C, D). In contrast, membrane ruffles markedly increased in WT macrophages exposed to apo­ptotic bodies. Activation of Rac1 with CN02 increased the levels of Rac1-GTP in both WT and SR-BI^−/−^ macrophages (Fig. 5A). Interestingly, CN02 treatment corrected the defective efferocytosis and membrane ruffling in SR-BI^−/−^ macrophages exposed to apoptotic thymocytes (Fig. 5B–D). The finding that pharmacological activation of Rac1 rescues efferocytosis in the absence of SR-BI supports the view that Rac1 is a mediator of efferocytosis from convergent signaling pathways (16). As CN02 likely activates multiple signaling pathways, we also examined the effects of the specific Rac1 inhibitor, NSC23766, on efferocytosis in WT versus SR-BI^−/−^ macrophages (Fig. 5E, F). NSC23766 markedly decreased the levels of active Rac1 in WT cells (Fig. 5G) and reduced WT efferocytosis of apoptotic thymocytes to levels similar to SR-BI^−/−^ cells (Fig. 5E, F). In addition, NSC23766 did not affect SR-BI^−/−^ macrophage efferocytosis, suggesting that Rac1 activation in efferocytosis is downstream of SR-BI. We also examined potential involvement of efferocytosis-related factors in SR-BI-deficient macrophages; we did not find significant changes of several relevant genes or proteins, including *Mertk*, *C1qa*, *C1qc*, *Gas6*, *Mfge8*, *Thbs1*, *annexin A*, *CD36* and *CD68*, and* Arg-1* and *iNos* (supplementary Fig. 7A, B).

**Fig. 6.**
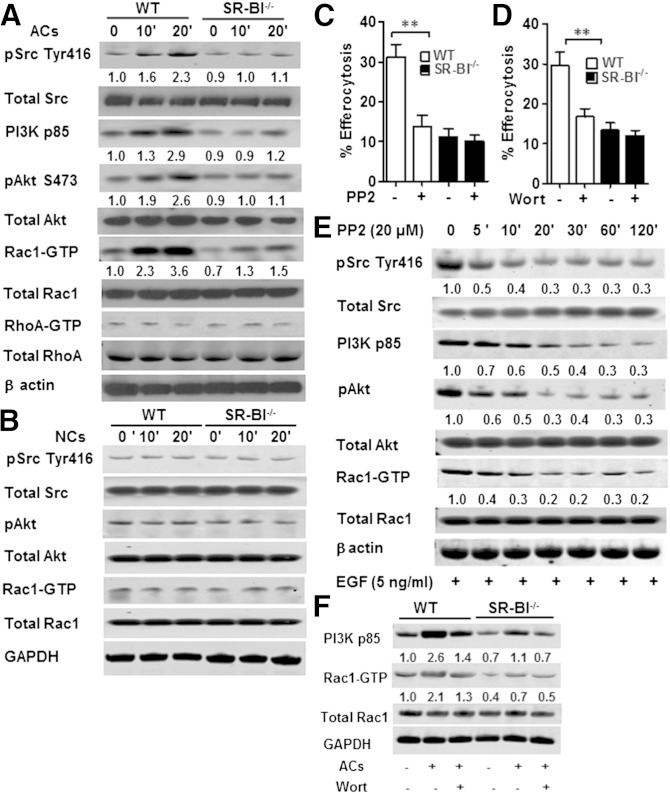
SR-BI mediates efferocytosis via a Src/PI3K/Rac1 signaling pathway. A: Western blot of pSrc (Tyr416), total Src, PI3K p85, pAkt (Ser473), total Akt, Rac1-GTP, total Rac1, RhoA-GTP, total RhoA, and β-actin in WT and SR-BI^−/−^ macrophages incubated alone or in the presence of apoptotic thymocytes (ACs) for 10 or 20 min. B: Western blot of pSrc, total Src, pAkt, total Akt, Rac1-GTP, total Rac1, and GAPDH in WT and SR-BI^−/−^ macrophages incubated with or without necrotic cells (NCs) at the indicated time points. C, D: WT and SR-BI^−/−^ macrophages were incubated with either the Src inhibitor, PP2 (C) (20 μM) or the PI3K inhibitor, Wortmannin (D) (30 nM) and the uptake of apoptotic thymocytes was quantitated as described in the Materials and Methods. The data are presented as mean ± SEM of three experiments. **P < 0.01. E: Western blot of pSrc (Tyr416), total Src, PI3K p85, pAkt (Ser473), total Akt, Rac1-GTP, total Rac1, and β-actin in WT macrophages incubated with 20 μM PP2 at the indicated times. F: Western blot of PI3K p85, Rac1-GTP, total Rac1, and GAPDH in macrophages incubated with apoptotic thymocytes in the absence and presence of Wortmannin (30 nM). A, B, E, F: Western blots are representative and numbers are the mean of three experiments.

**Fig. 7.**
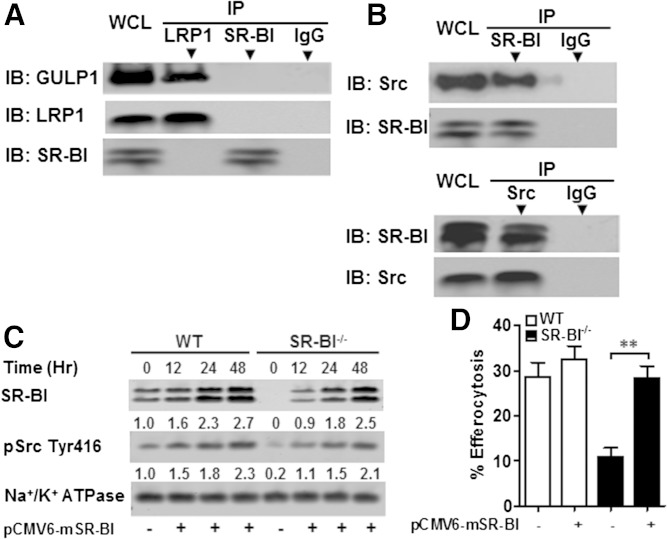
SR-BI interacts with Src in macrophages, inducing Src phosphorylation and membrane recruitment of Src. A: WT macrophage cell lysates were immunoprecipitated with either anti-SR-BI or anti-LRP1 antibodies and protein A/G magnetic beads. Immunoprecipitated GULP or SR-BI as a control was then detected by Western blotting. B: WT macrophage cell lysates were immunoprecipitated with either anti-SR-BI or anti-Src antibodies and protein A/G magnetic beads. Immunoprecipitated Src or SR-BI was then detected by Western blotting. C: SR-BI plasmid was transfected for the indicated times into WT or SR-BI^−/−^ macrophages using Lipofectamine LTX and Plus reagent. Plasma membrane proteins were isolated as described in the Materials and Methods and the SR-BI, pSrc (Tyr 416), and Na^+^/K^+^ ATPase levels were detected by Western blotting. D: Efferocytosis was measured by flow cytometry from WT and SR-BI^−/−^ macrophages transfected with pCMV6-mSR-BI plasmid or sham as described in the Materials and Methods. A–C: Data are representative of three experiments.

Studies have shown that Src activation induces Rac1 activity (17) and that PI3K activity is important for phagosome formation via the Fcγ receptor (18). Therefore, we examined the possibility that macrophage SR-BI mediates efferocytosis by activating Rac1 via a Src/PI3K pathway (**Fig. 6**). Compared with SR-BI^−/−^ macrophages, incubation of WT macrophages with apoptotic thymocytes markedly enhanced the activation of Src as evidenced by increased phosphorylation of tyrosine 416 in the kinase catalytic domain of Src (Fig. 6A). SR-BI also induced activation of PI3K, as shown by increased PI3K p85 and pAkt levels in WT versus SR-BI^−/−^ macrophages exposed to apoptotic cells (Fig. 6A). Following activation of Src, WT macrophage Rac1 activity was strikingly induced, but RhoA activity, which has been shown to inhibit efferocytosis, was not altered (Fig. 6A). Both Src and PI3K activation were critical to SR-BI-mediated efferocytosis, as inhibition with PP2 or Wortmannin, respectively, decreased efferocytosis in WT macrophages but had no effect in SR-BI^−/−^ cells (Fig. 6C, D). In addition, incubation with necrotic cells did not induce activation of Src, PI3K, and Akt in WT cells (Fig. 6B), demonstrating that the signaling is specific for efferocytosis of apoptotic cells. SR-BI-induced Src phosphorylation must act upstream of PI3K/Rac1 activation, as inhibition of Src phosphorylation with PP2 decreased PI3K p85, pAkt, and Rac1-GTP levels in WT macrophages (Fig. 6D). Rac1 was confirmed to be a downstream target of PI3K, as inhibition of PI3K by Wortmannin decreased Rac1-GTP levels in WT cells (Fig. 6F). In contrast to observations with Sertoli cell SR-BI (19) or macrophage LDL receptor-related protein 1 (LRP1), macrophage SR-BI was not associated with the engulfment adaptor PTB domain (GULP) (**Fig. 7A**). Importantly, coimmunoprecipitation of SR-BI and Src demonstrated that the two proteins are directly associated in WT macrophages (Fig. 7B), suggesting that SR-BI plays a direct role in activation of Src in macrophages. Several receptors cause Src membrane targeting and subsequent autophosphorylation (20). Therefore, we examined the effects of SR-BI expression on plasma membrane recruitment of activated Src by transfecting WT or SR-BI^−/−^ macrophages with pCMV6-mSR-BI plasmid. In both cell types incubated with apoptotic cells, plasma membrane pSrc levels increased in a stepwise fashion over time with increasing levels of SR-BI expression (Fig. 7C), demonstrating that SR-BI activates Src in macrophages.

Recent studies have shown that the proinflammatory necrotic cell marker, HMGB1, binds cytoplasmic Src, preventing its association with transmembrane receptors (21). Compared with WT cells isolated from recipient LDLR^−/−^ mice fed a Western diet, SR-BI^−/−^ macrophages had 2- and 1.7-fold higher HMGB1 mRNA and serum protein levels, respectively (supplementary Fig. 8A, C). In addition, HMGB1 protein levels were increased in SR-BI^−/−^ cells compared with WT macrophages with and without free cholesterol enrichment (supplementary Fig. 8B). Consistent with SR-BI regulating HMGB1 levels, transfection of SR-BI^−/−^ macrophages with SR-BI markedly decreased HMGB1 protein (supplementary Fig. 8D). Interestingly, knockdown of HMGB1 expression partially restored efferocytosis in SR-BI^−/−^ macrophages, suggesting that SR-BI regulation of HMGB1 also impacts Src activation and efferocytosis via other receptors (supplementary Fig. 8E).

## DISCUSSION

Our studies examined the hypothesis that macrophage SR-BI plays a critical role in mediating efferocytosis of apoptotic cells in atherosclerotic lesions. Our findings demonstrate that macrophage SR-BI binds PS on apo­ptotic cells and mediates efferocytosis via a Src/PI3K/Rac1 pathway. Hematopoietic SR-BI deletion resulted in dramatically increased atherosclerotic lesion necrosis and noninternalized dead cells with reduced collagen content and fibrous cap thickness, supporting a critical role for macrophage SR-BI in mediating efferocytosis in vivo. Combined deletion of macrophage SR-BI and ApoE exacerbated the impairment in efferocytosis and the accumulation of lesion-free dead cells beyond the single deletion of either gene, suggesting that SR-BI mediates efferocytosis by processes that are both dependent and independent of its interaction with ApoE.

### Effects of macrophage SR-BI on atherosclerotic lesion development, efferocytosis, and necrosis

Similar to previous studies by us and others (5, 6), we found that hematopoietic cell deficiency of SR-BI accelerates atherosclerosis development in ApoE^−/−^ and LDLR^−/−^ mice. Defective efferocytosis is believed to accelerate atherosclerosis and promote plaque vulnerability by causing increased inflammation and necrosis in atherosclerotic lesions (8, 22). Here we demonstrate, for the first time, that SR-BI is localized in phagosomes of WT macrophages and can directly bind PS in vitro and at the surface of apoptotic macrophages (supplementary Fig. 6), and that the efferocytosis of apoptotic cells by SR-BI-deficient macrophages is decreased both in vitro and in vivo (Fig. 1). Similarly, Kawasaki et al. (19) demonstrated that Sertoli cell SR-BI binds PS liposomes. We clearly show that hematopoietic SR-BI deficiency leads to a marked accumulation of lesion-free apoptotic cells and enlarged necrotic core formation, which are hallmarks of defective efferocytosis and lesion progression in vivo. Hence, our studies suggest that a major atheroprotective mechanism mediated by macrophage SR-BI is the efferocytosis of apoptotic cells.

Several studies led to our hypothesis that macrophage SR-BI expression plays a critical role by mediating lesion efferocytosis. Rat Sertoli cells can mediate phagocytosis of apoptotic spermatogenic cells via SR-BI (19, 23), and transgenic expression of human SR-BI in HEK-293 cells promotes engulfment of apoptotic thymocytes (24). Our current results show, for the first time, that SR-BI plays a critical role in mediating efferocytosis of apoptotic cells in macrophages, which are professional phagocytes. Importantly, the number of free dead cells was markedly increased in atherosclerotic lesions containing SR-BI^−/−^ macrophages (Fig. 3). Both enhanced apoptosis and defective efferocytosis contribute to formation of the necrotic core. We observed that SR-BI deficiency also led to increased macrophage apoptosis in atherosclerotic lesions (supplementary Fig. 5), but our data suggests that SR-BI plays a major role in the efferocytosis of apoptotic cells in lesions containing DKO cells, as the effects on active caspase 3 levels were modest compared with the accumulation of free dead cells. If apoptotic cells are not cleared quickly, they undergo secondary necrosis, release inflammatory cytokines, and elicit immune responses (7). The physiological relevance of macrophage SR-BI-mediated efferocytosis in atherosclerosis is supported by the tremendous increase in plaque necrosis. After only 8 weeks on a Western diet, plaque necrosis was increased 4-fold and the ratio of free to macrophage-associated TUNEL stain was up 14-fold in recipients of DKO marrow compared with controls (Figs. 3, 4). A key feature of vulnerable plaque is collagen degradation. We demonstrated that hematopoietic SR-BI deficiency dramatically reduced the collagen and fibrous cap thickness in lesions of DKO→ApoE^−/−^ mice compared with ApoE^−/−^→ApoE^−/−^ mice (Fig. 4). Consistent with this concept, hematopoietic SR-BI deficiency resulted in increased expression of the proinflammatory markers IL-1β, IL-6, MCP-1, MMP-9, and TNF-α, and decreased expression of the anti-inflammatory cytokines IL-10 and TGF-β (Fig. 2). These observations clearly reveal that hematopoietic cell SR-BI deletion elicits maladaptive inflammatory responses that are associated with defective efferocytosis and contribute to atherosclerosis development. Other studies have shown that SR-BI-null mice develop a systemic autoimmune response characterized by autoantibodies and deposition of renal immune complexes (25). SR-BI deficiency also enhances lymphocyte proliferation (25). However, we determined that plaque lymphocyte content was not affected by hematopoietic SR-BI deficiency (supplementary Fig. 9), but that the live macrophage content was decreased (supplementary Fig. 3C), suggesting that dying macrophages are the key inflammatory cells impacting atherogenesis in SR-BI^−/−^ lesions.

Our studies suggest that ApoE also protects against atherosclerosis by controlling efferocytosis, as deletion of hematopoietic ApoE in LDLR^−/−^ mice caused lesional accumulation of free dead cells (Fig. 3). In vitro studies have shown that phagocyte and apoptotic cell ApoE enhance efferocytosis (26, 27), and ApoE binds PS (data not shown). Furthermore, ApoE binds SR-BI to stimulate selective cholesteryl ester uptake and cholesterol efflux, suggesting a functional interaction (28). However, our results show that combined deletion of hematopoietic SR-BI and ApoE in LDLR^−/−^ mice promotes the accumulation of lesion-free apoptotic cells, necrosis, and development of atherosclerosis beyond deletion of either gene alone (Figs. 3, 4), supporting independent anti-atherogenic roles. In addition, receptor-mediated efferocytosis is enhanced by PS bridging molecules (29) making it likely that SR-BI-mediated phagocytosis is made more efficient by other proteins, such as β2-glycoprotein 1 and MFGE8 (29).

### Macrophage SR-BI mediates efferocytosis via Src/PI3K/Rac1 signaling

Two overlapping signaling pathways have evolved to mediate efferocytosis in *Caenorhabditis elegans* and mammals: CED-1 (LRP1)/CED-6 (GULP)/CED-10 (Rac) and CED-2 (CrkII)/CED-5 (DOCK180)/CED-12 (ELMO)/CED-10 (Rac). Both pathways converge at Rac1 activation (30, 31). Osada and colleagues (32) have shown that the association of GULP with SR-BI is required for activation of MAPK and Rac1 in nonprofessional phagocytic Sertoli cells. In contrast, we found that macrophage SR-BI does not associate with GULP (Fig. 7A), showing that macrophage SR-BI efferocytosis signaling is uniquely different from the Sertoli cell SR-BI pathway. We report that signaling molecules downstream of SR-BI, including Src and PI3K, play critical roles in SR-BI-mediated induction of Rac1 activation. Indeed, incubation of SR-BI-expressing macrophages with apoptotic cells induces SR-BI/Src interaction, Src membrane recruitment, and Src phosphorylation (Fig. 7B, C). Importantly, inhibition of Src decreased efferocytosis and Rac1 activation in WT cells. The mechanisms for Src activation vary among different receptors and cell types. In endothelial cells, SR-BI-induced activation of Src resulting in Rac1 activation requires association of the PDZK1 adaptor protein with the PDZ binding domain in the C terminal of SR-BI (33). However, THP-1 macrophages do not express PDZK1 (34), and we did not detect PDZK1 mRNA in peritoneal macrophages (data not shown). Thus, interaction with the PDZK1 adaptor protein is likely not relevant for macrophage SR-BI efferocytosis and suggests that macrophage and endothelial cell SR-BI activate Src via different mechanisms. PI3K activity is downstream of Src and critical to macrophage SR-BI-mediated efferocytosis, as inhibition of Src activity reduced pAkt and PI3K p85 levels, and inhibition of PI3K activity impaired efferocytosis in WT cells (Fig. 6). The PI3K products, pAkt and phosphatidylinositol 3,4,5-triphosphate [PI(3,4,5)P_3_], promote efficient efferocytosis (18) by mediating phagocyte survival (35) and PI(3,4,5)P_3_ and phagosome synthesis.

Macrophages have an abundance of efferocytic receptor pathways, and studies have shown that deletion of a single efferocytic receptor or bridging molecule (i.e., MFGE8, C1q) (7, 36) is sufficient to promote accumulation of free apoptotic cells in atherosclerotic lesions. These observations suggest that the environment of atherosclerotic lesions is proinflammatory and cytotoxic to the extent that multiple efferocytic pathways are required. Ligand interaction with both LRP1 (37) and Mertk (29) is associated with suppression of phagocyte toll-like receptor 4 inflammatory signaling. We also found that SR-BI-deficient phagocytes have a proinflammatory phenotype, which likely increases phagocyte death, further impairing efferocytosis. In addition, studies suggest that there is cross talk among efferocytic pathways making it likely that deletion of a critical player impacts phagocytosis via other receptors. Mertk-induced signaling enhances Rac1 activation and phagocytosis via αvβ5 integrin (38, 39). In addition, ligand interaction with LRP1 (40) regulates the internalization of phagocytic integrin αMβ2 (41). Because our results show that pharmacological activation of Rac1 corrects the defective efferocytosis in the absence of SR-BI (Fig. 5A, B), and specific inhibition of Rac1 significantly reduces efferocytosis in WT macrophages, but not in SR-BI-null macrophages (Fig. 5E–G), we propose that SR-BI-induced Src/PI3K/Rac1 signaling likely stimulates phagocytosis via other receptors as well. Consistent with this possibility, transfection of phagocytes with increasing amounts of SR-BI increased plasma membrane levels of pSrc Tyr416 in a stepwise dose response (Fig. 7C) and rescued defective efferocytosis from SR-BI-deficient macrophages (Fig. 7D). In addition, studies have shown that cytoplasmic HMGB1 interacts with Src, preventing association with plasma membrane receptors, which results in decreased Rac1 activation (21). These results raise the possibility that the effects of SR-BI on HMGB1 expression may impact other efferocytic pathways (supplementary Fig. 8).

In summary, macrophage SR-BI plays a crucial role in mediating efferocytosis of apoptotic cells in atherosclerosis, reducing plaque necrosis and inflammation. SR-BI interacts directly with apoptotic cell PS and induces efferocytosis signaling via Src/PI3K/Rac1 (supplementary Fig. 10). Besides directly mediating efferocytosis, SR-BI may also impact other efferocytic pathways by controlling Src activity via regulating HMGB1 levels, as well as by direct activation. Thus, macrophage SR-BI deficiency causes severely impaired lesion efferocytosis leading to secondary necrosis and a maladaptive inflammatory response (supplementary Fig. 10). Increased plaque necrosis due to defective efferocytosis may be a critical prelude to plaque rupture and myocardial infarction; hence, the signaling pathways involved in macrophage SR-BI-mediated efferocytosis provide attractive targets for plaque stabilization and the prevention of cardiovascular events.

## Supplementary Material

Supplemental Data
