# Supplementary material for: Macrophage SR-BI mediates efferocytosis via Src/PI3K/Rac1 signaling and reduces atherosclerotic lesion necrosis
Source: J Lipid Res. 2015 Aug;56(8):1449–60. doi: 10.1194/jlr.M056689 (PMC4513986; doi:10.1194/jlr.M056689)
Supplement: Supplemental Data [file supp_56_8_1449__index.html]

Macrophage SR-BI Mediates Efferocytosis via Src/PI3K/Rac1 Signaling and Reduces Atherosclerotic Lesion Necrosis — Macrophage SR-BI mediates efferocytosis via Src/PI3K/Rac1 signaling and reduces atherosclerotic lesion necrosis — Supplemental Data 

# Macrophage SR-BI mediates efferocytosis via Src/PI3K/Rac1 signaling and reduces atherosclerotic lesion necrosis

## Supplemental Data

- Supplemental Figures and Methods - Supplemental Data and Methods
