# Supplementary material for: Macrophage SR-BI mediates efferocytosis via Src/PI3K/Rac1 signaling and reduces atherosclerotic lesion necrosis
Source: J Lipid Res. 2015 Aug;56(8):1449–60. doi: 10.1194/jlr.M056689 (PMC4513986; doi:10.1194/jlr.M056689)
Supplement: Supplemental Data [file supp_M056689_jlr.M056689-1.pdf]

Supplemental Table

Table S1

Supplemental Table: Real time RT-PCR primer sequences used in this study

Gene name	Forward primer 5'-3'	Reverse primer 5'-3'
IL-1β	GGGCCTCAAAGGAAAGAATC	TACCAGTTGGGGAACTCTGC
IL-6	GGGCCTCAAAGGAAAGAATC	TACCAGTTGGGGAACTCTGC
TNF-α	TATGGCTCAGGGTCCAACTC	CTCCCTTTGCAGAACTCAGG
MCP-1	CCCAATGAGTAGGCTGGAGA	TCTGGACCCATTCCTTCTTG
IL-10	CCAAGCCTTATCGGAAATGA	TTTTCACAGGGGAGAAATCG
TGF-β	TTGCTTCAGCTCCACAGAGA	TGGTTGTAGAGGGCAAGGAC
MMP-9	CGTCGTGATCCCCACTTACT	GCTGTTGTGCTACACCAAGG
HMGB1	CCATTGGTGATGTTGCAAAG	CTTTTTCGCTGCATCAGGTT
β-actin	TGTTACCAACTGGGACGACA	GGGGTGTTGAAGGTCTCAAA
Gas6	CAAGACCTCATGGGCAACTT	AGCTTCCTGGTTTGTTGTGG
C1qa	CAGTGCCCGGCTTCTATTAC	CCCCTGCTAACACCTGAAAG
C1qc	TGTTCAACAGCAAGCAGGTC	CGGGAAACAGTAGGAAACCA
Thbs1	TATGTGCCTAATGCCAACCA	GCCATCACCATCAGAGTCCT
Annexin A1	TGTATCCTCGGATGTTGCTG	ATCCAAGGGCTTTCCATTCT
CD36	CCTTAAAGGAATCCCCGTGT	TGCATTTGCCAATGTCTAGC
CD68	ACATGGCGGTGGAATACAAT	TCAAGGTGAACAGCTGGAGA
Arg1	AAGCTGGTCTGCTGGAAAAA	ACACCAGCCAGCTCTTCATT
Nos2	CAAGCACCTTGGAAGAGGAG	AAGGCCAAACACAGCATACC
Mfge8	ATCTACTGCCTCTGCCCTGA	TGGCAGATGTATTCGGTGAA

Supplemental Figures

Fig. S1

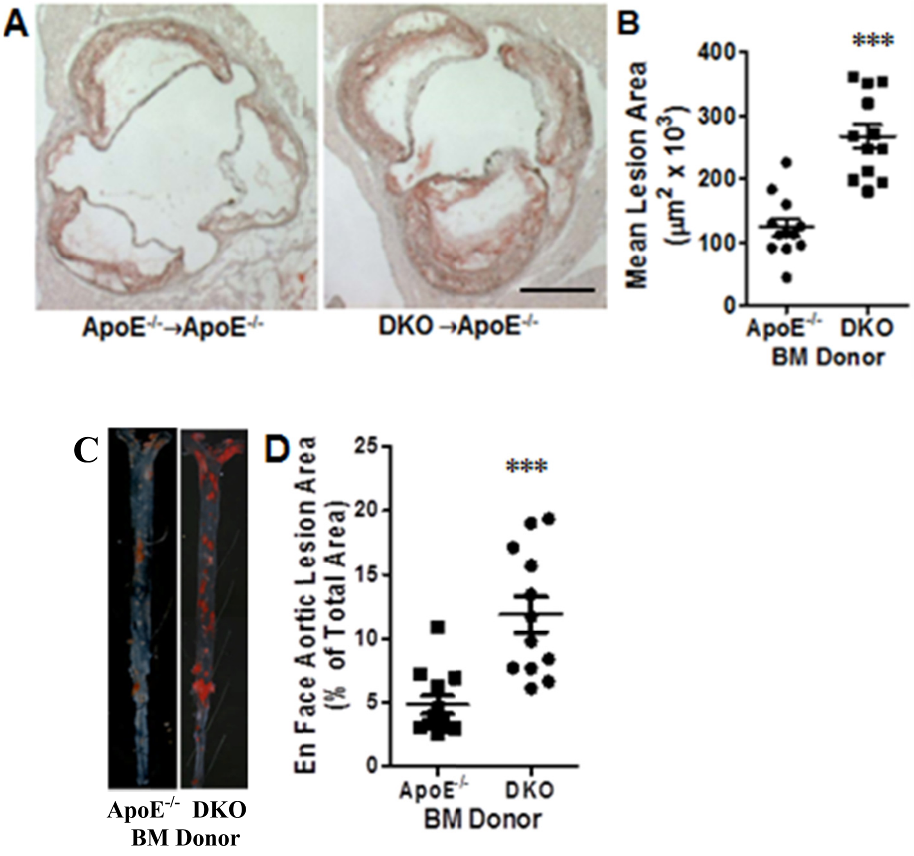

Fig. S1: Deletion of macrophage SR-BI accelerates atherosclerotic lesion development in BM recipient ApoE$^{-/-}$ mice. ApoE $^{-/-}$ female mice were reconstituted with ApoE$^{-/-}$ or DKO BM and fed a western diet for 8 weeks. (A and C) Representative images show Oil-Red-O stain in aortic root sections. Bar=200 µm (A) and in open-pinned aortas (C). (B and D) Quantitation of the mean Oil-Red-O stainable lesion area in aorta root sections (B) and en face aortas (D). N=12 per group, ***P<0.001.

Fig. S2

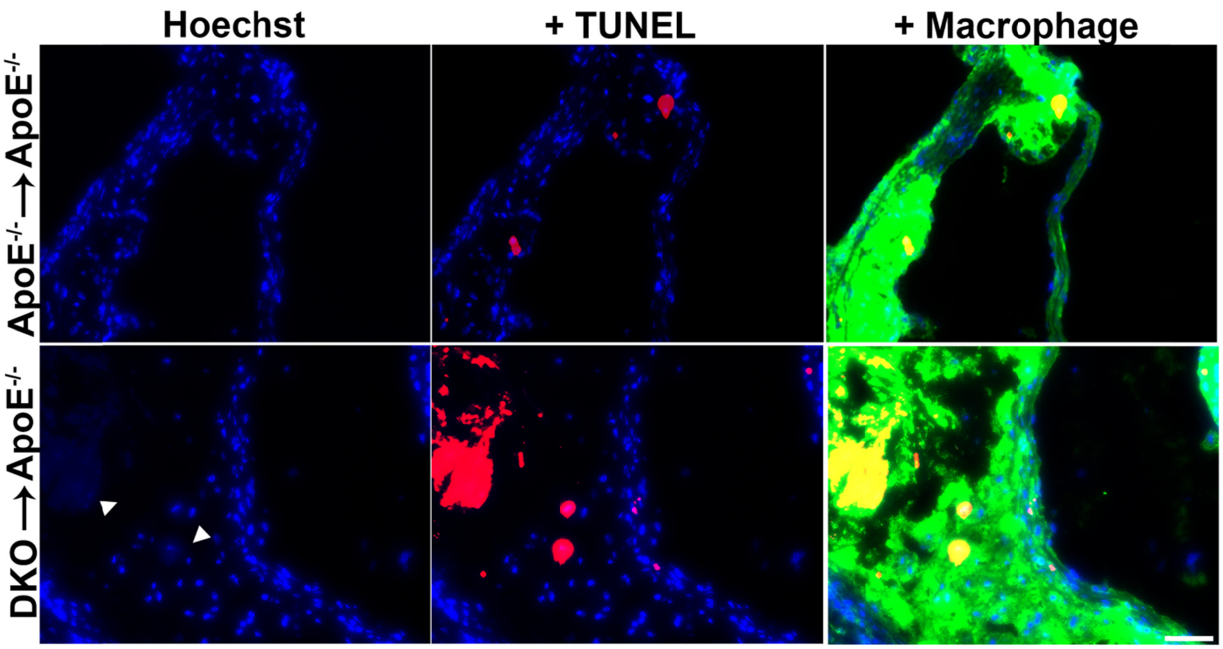

Fig. S2: Deletion of macrophage SR-BI promotes atherosclerotic lesion cell death in BM recipient ApoE$^{-/-}$ mice. Apoptotic cells were detected by TUNEL staining of proximal aorta sections from ApoE$^{-/-}$ mice reconstituted with ApoE $^{-/-}$ or DKO BM and fed a western diet for 8 weeks. Micrographs show images of nuclei (Hoechst, blue), TUNEL positive staining (red), and macrophages (green). Triangles point to fragmented, diffuse, and/or faded Hoechst stained nuclei. Bar=200 µm.

Fig. S3

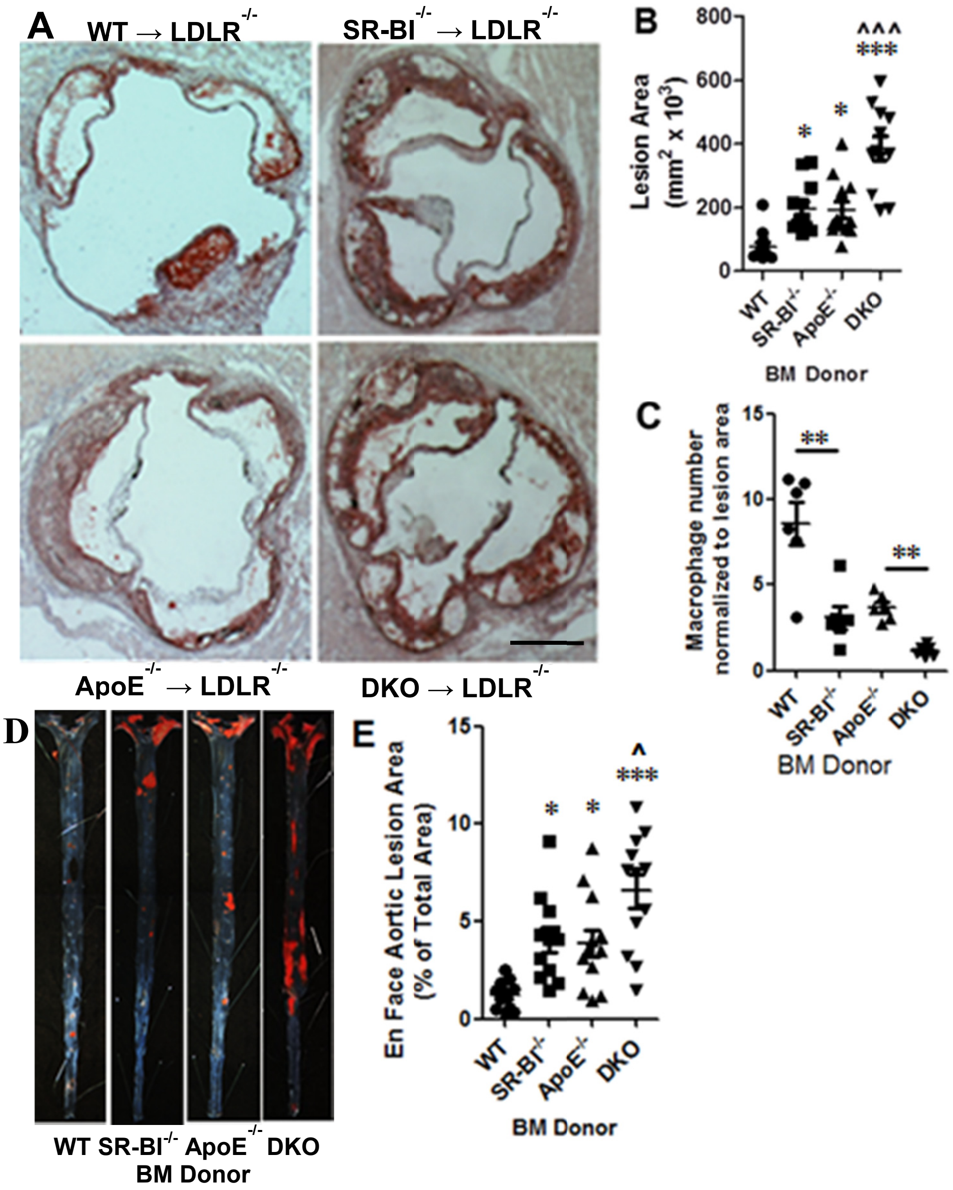

Fig. S3: Deletion of macrophage SR-BI promotes atherosclerotic lesion formation in BM recipient LDLR$^{-/-}$ mice. LDLR$^{-/-}$ female mice were reconstituted with WT, SR-BI$^{-/-}$, ApoE$^{-/-}$ or DKO BM and fed a western diet for 16 weeks. (A and D) Representative images show Oil-Red-O stain in aortic root sections (A) and in open-pinned aortas (D). (B and E) Quantitation of the mean Oil-Red-O stainable lesion in aortic root sections, bar=200 µm. (B) and en face aortas (E). (C) Quantitation of the number of live macrophages in aortic root sections. Macrophages were detected using rabbit anti-macrophage antibody and Alexa Fluor 488 labeled secondary antibody. Live macrophages were counted as macrophage stain surrounding Hoechst stained nuclei that were negative for TUNEL stain (see Figure 3 and Supplemental Figure 4). N=12 per group, *P<0.05, ***P<0.001 compared with WT, ^P<0.05 compared with either SR-BI$^{-/-}$ or ApoE$^{-/-}$.

Fig.S4

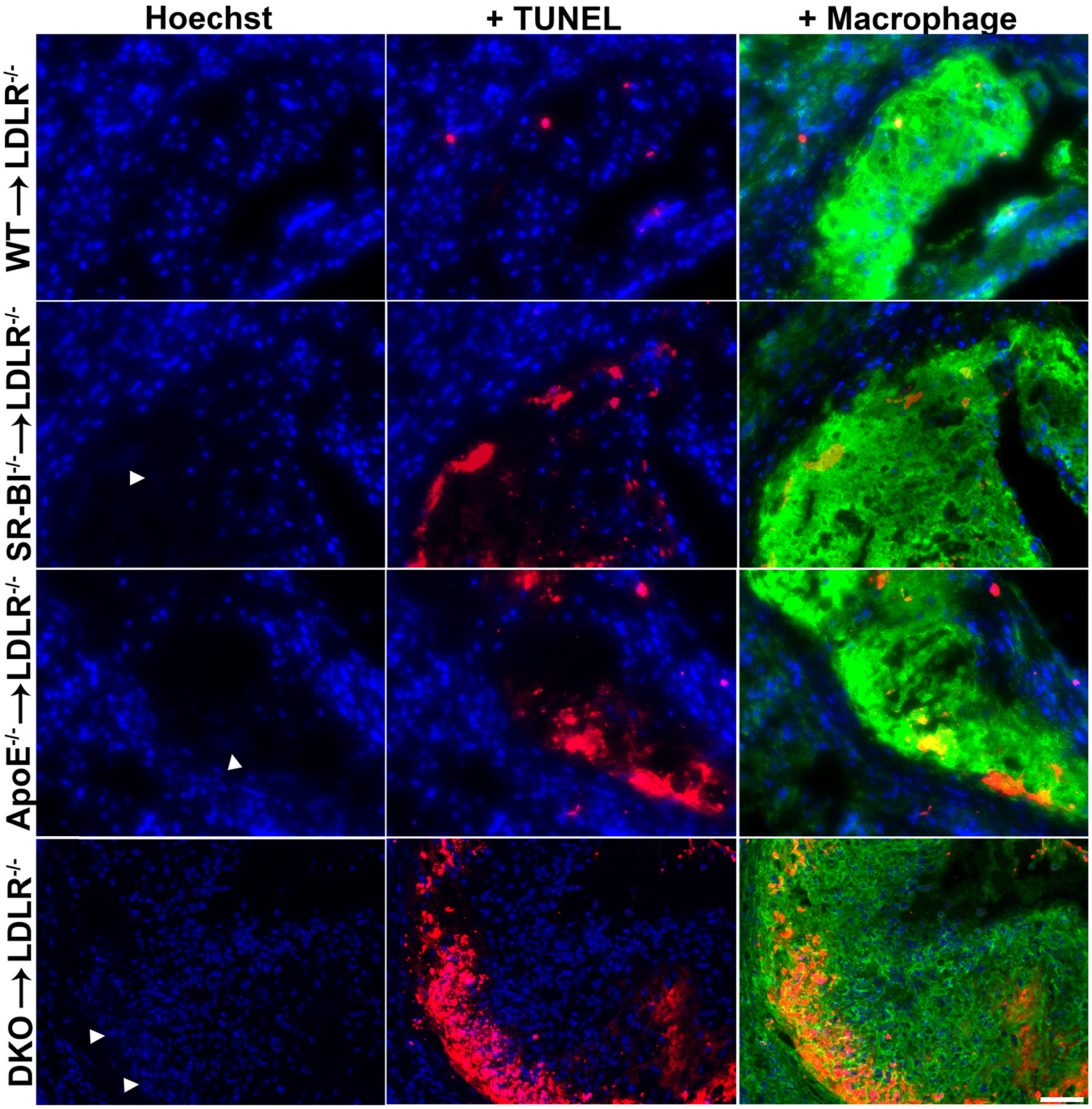

Fig. S4: Deletion of macrophage SR-BI promotes atherosclerotic lesion cell death in BM recipient LDLR$^{-/-}$ mice. Apoptotic cells were detected by TUNEL staining of proximal aorta sections from LDLR$^{-/-}$ mice reconstituted with WT, SR-BI$^{-/-}$, ApoE $^{-/-}$ or DKO BM and fed a western diet for 16 weeks. Micrographs show images of nuclei (Hoechst, blue), TUNEL positive staining (red), and macrophages (green). For the DKO lesion image, the TUNEL exposure was 10% of the exposure used for WT, SR-BI$^{-/-}$, and ApoE $^{-/-}$ lesion images. Triangles point to fragmented, diffuse, and/or faded Hoechst stained nuclei. Bar=200 μm.

Fig. S5

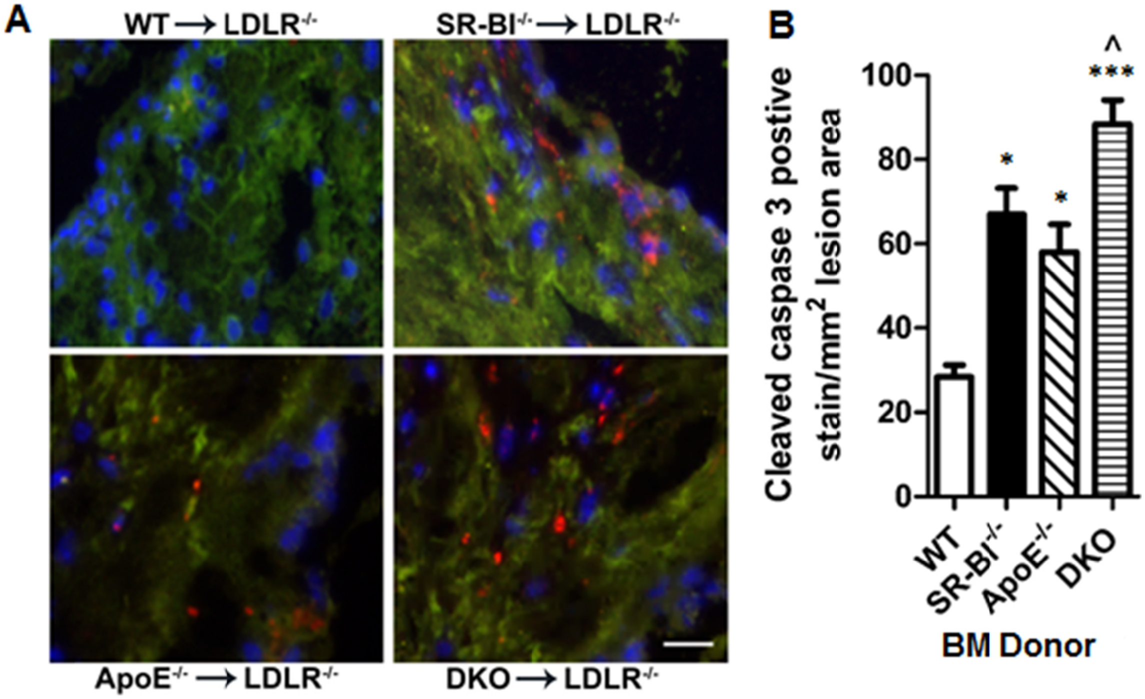

Fig. S5: **Hematopoietic SR-BI deletion causes increased macrophage apoptosis in proximal aorta sections of BM recipient LDLR$^{-/-}$ mice.** (A) Apoptotic cells were detected by cleaved caspase 3 staining of proximal aorta sections from LDLR$^{-/-}$ mice reconstituted with WT, SR-BI$^{-/-}$, ApoE $^{-/-}$ or DKO BM and fed a western diet for 16 weeks. Micrographs show images of nuclei (Hoechst, blue), cleaved casepase 3 positive staining (red), macrophages (green), bar = 200 µm. (B) Quantitation of cleaved caspase 3 positive stain in total lesion area. *P<0.05, **P<0.01, ***P<0.001 compared to WT; ^P<0.05 compared to singe SR-BI/ApoE knockout.

Fig. S6

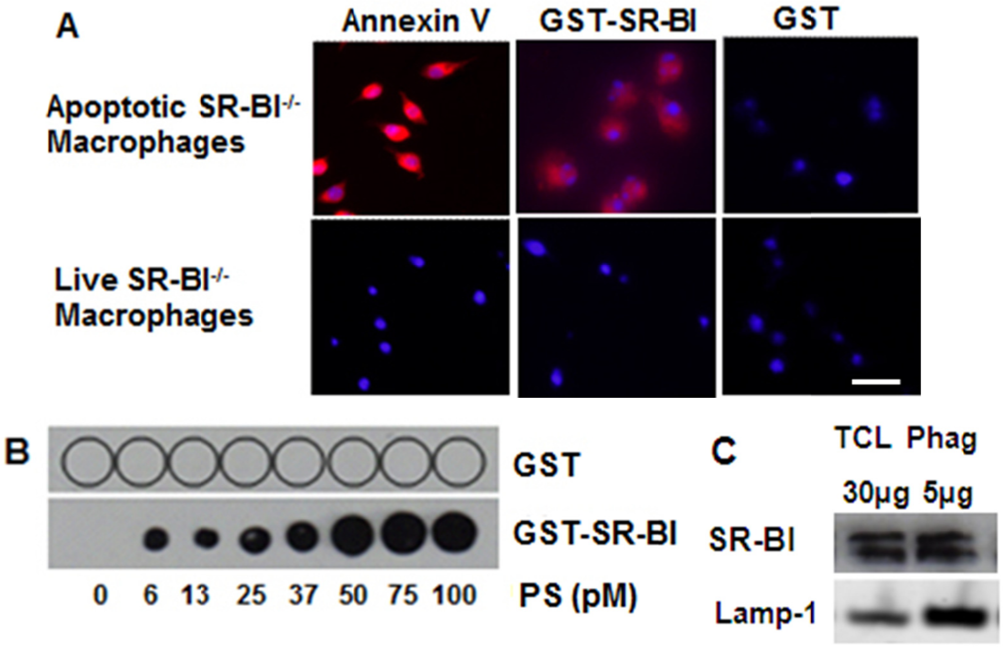

Fig. S6: SR-BI protein binds with phosphatidylserine (PS) *in vitro* and at the surface of apoptotic cells and localizes in phagosomes. (A) SR-BI$^{-/-}$ macrophages were incubated for 1h with BAY11-7082 (20 µmol/L) and exposure of PS was analyzed by Cy5 fluorescent labeled Annexin V directly or by incubation with GST-tagged SR-BI protein or GST alone for control. SR-BI was then detected using anti-SR-BI antibody and an Alexa 568 fluorescence labeled secondary antibody. The cells were fixed in 4% paraformaldehyde, counterstained with DAPI, visualized using fluorescence microscopy. Micrographs show Annexin V or SR-BI positive cells (Red) and nuclei (Blue), bar=50 µm. (B) Increasing concentrations of PS were loaded onto the nitrocellulose membrane and incubated for 1 hour with 1 µg/ml GST tagged SR-BI or GST alone for control. Bound protein was detected by anti-SR-BI and secondary antibody. (C) Phagocytosis was stimulated with latex beads and phagosomes isolated as described in Methods. SR-BI and Lamp-1 in phagosomes (5 µg protein) and total cell lysate (30 µg protein) were detected by Western blot. (A-C) Data are representative of 3 experiments.

Fig. S7

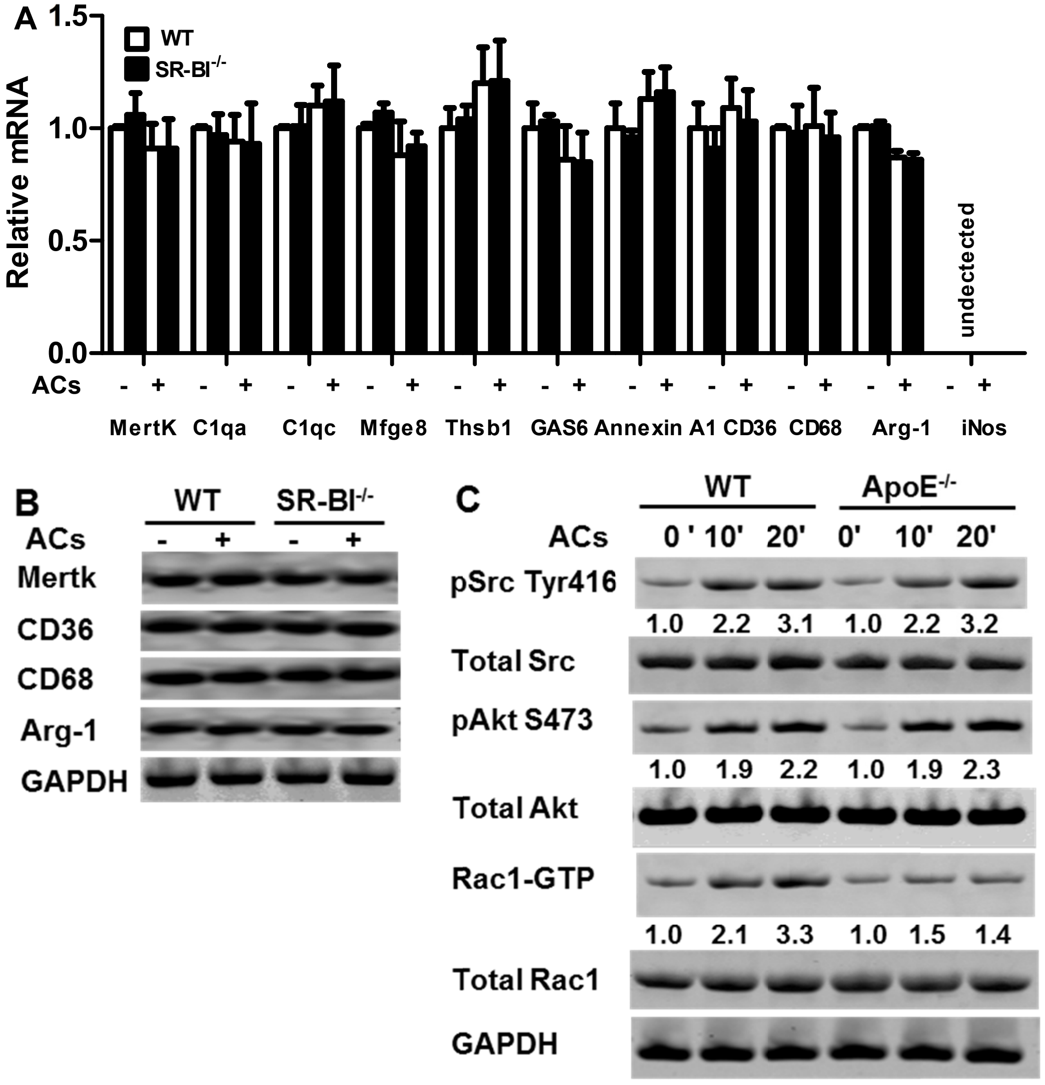

Fig. S7: The role of SR-BI and ApoE in regulation of efferocytosis signals. WT and SR-BI$^{-/-}$ peritoneal macrophages were treated with or without apoptotic cells (ACs) for 12 hours, the indicated efferocytosis-related genes or proteins were measured by real time RT-PCR (A) or Western blot (B). Western blot of pSrc, total Src, pAkt, total Akt, Rac1-GTP, total Rac1 and GAPDH from whole cell lysates made from WT and ApoE$^{-/-}$ peritoneal macrophages treated with or without ACs at indicated time points. Western blots are representative and numbers are the mean of 3 experiments (C).

Fig. S8

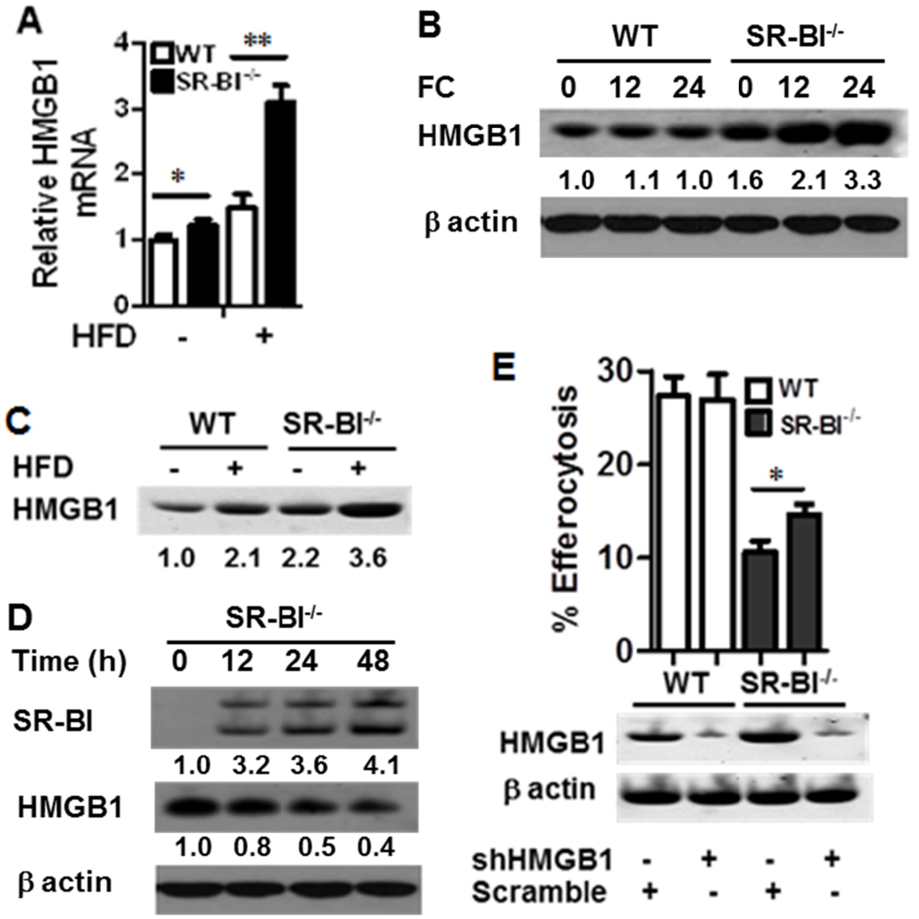

Fig. S8: Deletion of macrophage SR-BI affects HMGB1 expression. (A) HMGB1 mRNA levels were determined by real-time RT-PCR in macrophages isolated from $LDLR^{-/-}$ mice transplanted with WT or $SR\text{-}BI^{-/-}$ BM and fed either chow or high fat diet (HFD) for 16 weeks. The data are presented as mean ± SEM of 3 experiments with statistical difference between WT and $SR\text{-}BI^{-/-}$ levels indicated by *P<0.05, **P<0.01. (B) Western blot of HMGB1 in WT and $SR\text{-}BI^{-/-}$ macrophages incubated with 100 μg/ml of acetylated LDL and an ACAT inhibitor (58035, 10 μg/ml) at the indicated times. Quantitation is the average of 3 experiments. (C) Western blot of serum HMGB1 from WT and $SR\text{-}BI^{-/-}$ mice fed with or without high fat diet (HFD). The quantitation is the average of 6 mice per group. (D) SR-BI plasmid was transfected for the indicated times into $SR\text{-}BI^{-/-}$ macrophages using Lipofectamine LTX and Plus reagent. HMGB1, SR-BI, and β-actin were then detected by western blotting. (E) Knockdown of HMGB1 by lentiviral expression of HMGB1 shRNA in macrophages lacking SR-BI promoted efferocytosis. Data are mean ± SEM of 3 experiments. *P<0.05.

Fig. S9

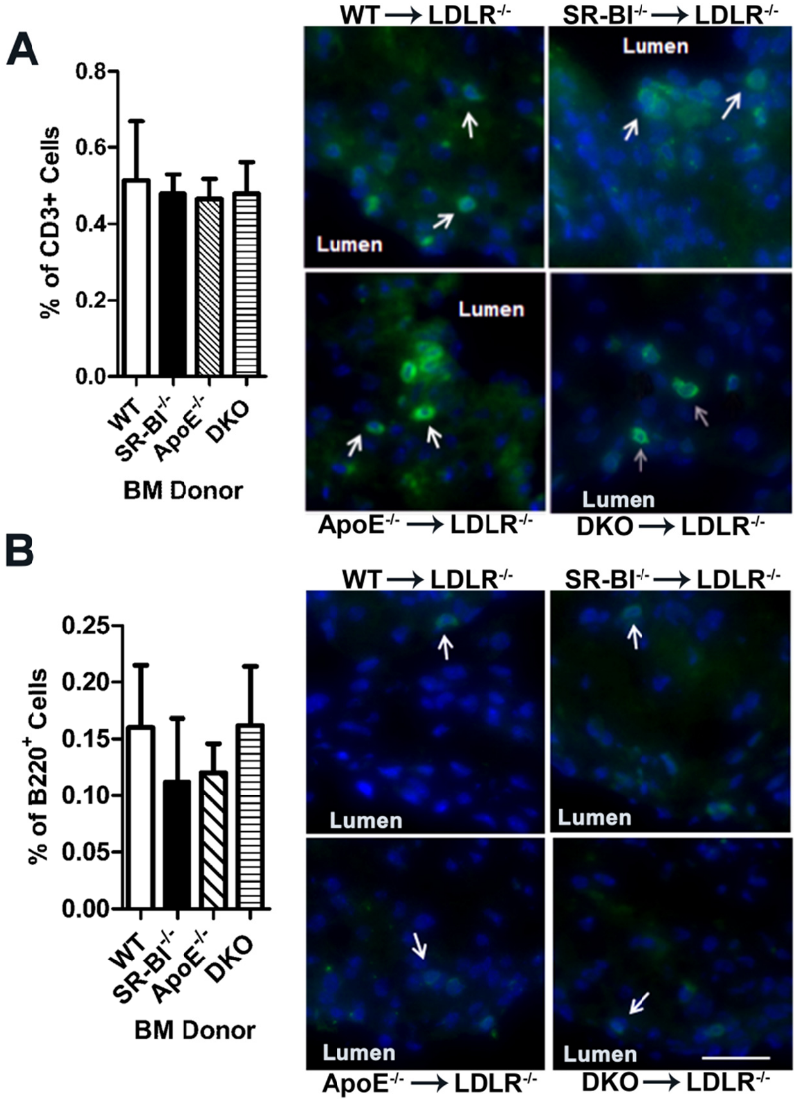

Fig. S9: Deletion of SR-BI in bone marrow does not alter plaque lymphocyte content. The T and B lymphocyte content in the atherosclerotic lesions of LDLR$^{-/-}$ mice reconstituted with WT, SR-BI$^{-/-}$, ApoE$^{-/-}$ and DKO BM and fed a Western diet for 16 weeks. The **T** (A) and B (B) lymphocytes were detected with anti-CD3 and B220 antibodies and FITC-labeled secondary antibodies, respectively. The percent of CD3 and B220 positive cells of total lesion cells were analyzed by ImageJ software. (A and B), N=6, each group, No statistical differences among WT, SR-BI$^{-/-}$, ApoE$^{-/-}$ and DKO groups. Shown are representative images from each group. Green, T cells (A) or B cells (B), blue, nuclei, bar=50 μm.

Fig. S10

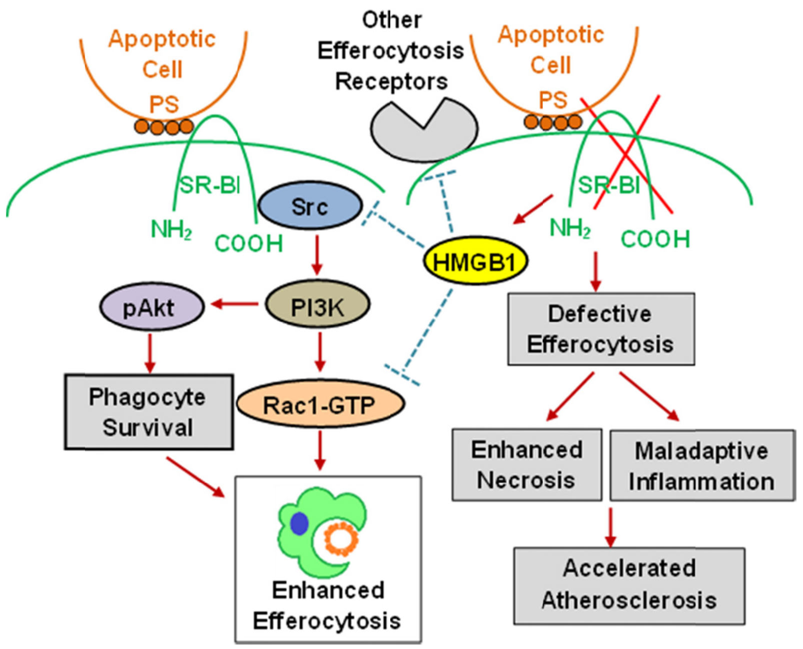

Fig. S10: Proposed mechanism of macrophage SR-BI in regulation of efferocytosis. Macrophage SR-BI directly mediates efferocytosis by binding apoptotic cell phosphatidylserine and inducing signaling via Src/PI3K/Rac1. Macrophage SR-BI also indirectly regulates other efferocytosis pathways and signaling by regulating HMGB1 expression. Thus, macrophage SR-BI deficiency promotes severely impaired efferocytosis resulting in enhanced necrosis and maladaptive inflammation.

Supplemental Methods

Mice: SR-$BI^{+/-}$ (1:1 mixed C57BL/6 X S129 genetic background) and $ApoE^{-/-}$ mice on a C57BL/6 background were obtained from The Jackson Laboratory (Bar Harbor, ME). SR-$BI^{+/-}$ (1:1 mixed C57BL/6 X S129 genetic background) were backcrossed over 10 generations onto the C57BL/6 background. SR-$BI^{+/-}ApoE^{-/-}$ mice were then mated to produce SR-$BI^{-/-}ApoE^{-/-}$ mice on the C57BL/6 background. Animal care and experiment protocols were carried out according to the regulations of Vanderbilt University's Institutional Animal Care and Usage Committee. Mice were maintained on rodent chow diet or fed a Western-type diet containing 21% milk fat and 0.15% cholesterol (Teklad, Madison, WI).

Atherosclerosis analyses: Eight-week-old female $ApoE^{-/-}$ mice were lethally irradiated by 9 Gy from a cesium gamma source and transplanted with 5×10^6 BM cells from SR-$BI^{+/+}$ $ApoE^{-/-}$ and SR-$BI^{-/-}$ $ApoE^{-/-}$ (DKO) mice (1). After 4 weeks, the mice were placed on western-type diet for 8 weeks. To compare the four genotypes of macrophages, recipient 8-week-old female $LDLR^{-/-}$ mice were irradiated and transplanted with BM from WT, SR-$BI^{-/-}$, $ApoE^{-/-}$, and DKO mice and maintained on a western-type diet for 16 weeks. The extent of atherosclerosis was examined both in Oil-Red-O stained cross-sections of the proximal aorta (15 alternate 10-μm cryosections) and by *en face* analysis using the KS300 imaging system (Kontron Elektronik GmbH) (1).

Cell culture and transfections: Thioglycollate-elicited peritoneal macrophages were isolated (2) from mice reconstituted with WT, SR-$BI^{-/-}$, $ApoE^{-/-}$ and DKO BM. Mouse thymocytes were isolated as described (3). For transient transfections, 3 μL Lipofectamine LTX and 2 μL Plus reagent (Invitrogen) with 1 μg of pCMV6-mSR-BI plasmid (OriGene Technologies) were applied to 1×10^6 macrophages. For gene knockdown assay, WT and SR-$BI^{-/-}$ peritoneal macrophages were infected with shHMGB1 or scramble shRNA lentivirus (Origene Technologies) using the manufacturer's protocol.

Analyses of efferocytosis and membrane ruffling: For in vitro efferocytosis assays, WT thymocytes were labeled with CFDA-SE green cell tracer (Invitrogen) and apoptosis was induced with 600 rad irradiation. Apoptotic cells were added onto phagocytes at a ratio of 10:1, and incubated for 2h. After vigorous washing with PBS, the efferocytosis of apoptotic cells was then visualized using fluorescence

microscopy and quantitated by flow cytometry (4, 5). For *ex vivo* efferocytosis assays, $LDLR^{-/-}$ mice reconstituted with WT, $SR\text{-}BI^{-/-}$, ApoE-/- and DKO BM were injected with 3% thioglycollate. On day 3, 1 x 10^9 CFDA-SE labeled apoptotic thymocytes were injected into the peritoneal cavity. After 2h, cells were collected by peritoneal lavage, plated onto wells, stained with CMTPX red cell tracer (Invitrogen), and after 1h adherence, the macrophages were scraped and the percent of phagocytes containing apoptotic bodies was quantitated by flow cytometry (4, 5). For analysis of membrane ruffling, phagocytes were incubated for 20 min in the absence or presence of apoptotic thymocytes. After washing away apoptotic cells, the cells were then fixed, permeabilized, stained with rhodamine phalloidin (Cytoskeleton, Inc.), and counterstained with Hochest. The % phagocytes containing membrane ruffles as detected by concentrated punctuate regions of rhodamine phalloidin stained F-actin was quantitated using fluorescence microscopy. To examine the role of various signaling molecules in efferocytosis and membrane ruffling, cells were incubated with 0.1 μM PP2 (Src inhibitor, Sigma), 30 nM Wortmannin (PI3K inhibitor, Sigma) or 1 U CN02 (Rac1 activator, Cytoskeleton, Inc.) for the indicated time points.

Analyses of lesion necrosis, collagen content, fibrous cap thickness, cell death, and efferocytosis: Necrosis was detected using Harris's hematoxylin and eosin (H&E), and quantitated by measuring the acellular area in the intima versus total intimal area. For analysis of the collagen content and fibrous cap thickness in atherosclerotic lesions, aorta cryosections were stained with Masson's trichrome stain kit (Sigma), blue collagen area was qualified using ImageJ software as percent of total lesion area (6) and fibrous cap thickness was measured by ImageJ software as previous reported (6). For analysis of cell death and efferocytosis, 5-μm proximal aortic cryosections were stained with TUNEL using the TMR red (Roche) detection kit followed by manufacture's instruction. Briefly, the sections were fixed with fresh 4% paraformaldehyde, permeabilized with fresh permeabilization buffer containing 0.1% Triton X-100 in 0.1% sodium citrate, stained with TUNEL TMR red for 1 hour at 37°C, then blocked and incubated with rabbit anti-macrophage antibody (Accurate Chemical and Scientific Corp.), goat anti-rabbit Alexa Fluor 488 conjugated secondary antibody (Molecular Probes, Inc.). Nuclei were counterstained with Hoechst. The TUNEL positive nuclei were quantitated and normalized to total lesion area. In lesions where the

TUNEL stain was intense and blanketed areas where the Hoechst staining of nuclei was condensed, fragmented, and/or faded, the average area of healthy Hoechst-stained nuclei was used to quantitate TUNEL positive nuclei in the TUNEL stained area. The free versus macrophage-associated TUNEL stain in the same sections were quantitated as described (5) and expressed as the ratio of free to macrophage-associated dead cells. TUNEL stained nuclei were counted as free when they were not associated with or in close proximity to viable macrophages that were detected as Alexa Fluor 488 stained macrophage cytoplasm surrounding a Hoechst-stained nucleus.

Analysis of lesion lymphocyte content: Aorta cryosections were fixed with cold acetone, and blocked using Background Buster (Innovex Biosciences). T and B cells were detected using FITC-labeled anti-CD3 (BD Pharmingen) and FITC-labeled anti-B220 (BD Pharmingen), respectively. The percent of total cells positive for CD3 or B220 were then counted.

Phospholipid-SR-BI Binding Assay: Serial concentrations of PS (Avanti) were loaded onto nitrocellulose membrane-C (HyBond) (7), and incubated with GST-tagged SR-BI (Novus) or GST alone. After washing away unbound protein, the bound protein was detected by anti-SR-BI (Novus) or anti-GST (Novus) and secondary antibody (Sigma). For a cell-based assay, apoptosis was induced in SR-BI$^{-/-}$ macrophages by 20 μM BAY11-7082 (Sigma), and exposure of PS was analyzed by fluorescence labeled Annexin V directly or by incubation with GST-SRBI protein or GST alone. SR-BI was detected using anti-SR-BI antibody and a fluorescent-labeled secondary antibody. The cells were fixed in 4% paraformaldehyde, counterstained with Hoechst, and visualized using fluorescence microscopy.

Immunoprecipitation, Western Blotting, and Rac1-GTP activity Assay: Macrophage lysates (500 μg protein) and 10 μg of antibody against either SR-BI (Novus) or Src (Cell Signaling) were incubated overnight at 4°C and then incubated for 1 h at 4°C with 50 μl of A/G beads (Invitrogen). The magnetic beads were collected, washed, boiled with sample buffer at 70°C for 5 min, and the supernatant was used for detecting Src or SR-BI by immunoblotting. For western blot, primary antibodies specific for SR-BI (Novus), Akt and p-Akt, PI3K p85, HMGB1, Src and p-Src, Na^+/K^+-ATPase (Cell Signaling), Rac1 (Upstate), active Rac1-GTP (NewEast Biosciences), Lamp-1 (Millpore) and β-actin (Sigma), as well as

anti-rabbit/goat horseradish peroxidase-conjugated (Sigma) or fluorescent tagged (LI-COR) secondary antibody were applied. To assess activation of Rac1, aliquots of the cell extracts were loaded with GTPγS, immunoprecipitated with PAK1-PBD-agarose at 4°C for 60 minutes, and immunoblotted for Rac1 following the manufacturer's protocol (Millipore). Western blot band density was quantified by Quantity One software (BioRad 4.5.2 version).

Plasma Membrane Protein and Phagosome Preparation: For plasma membrane protein extraction, cells were scraped, pelleted, washed with cold PBS, and resuspended in 1 mL of the Homogenization Buffer Mix (Plasma Membrane Protein Extraction Kit, BioVIsion) and homogenized on ice. After centrifugation at 700 × g for 10 min at 4°C, the supernatant was collected and centrifuged at 10,000 × g for 30 min at 4°C. The membrane protein pellet was then dissolved in 0.5% Triton X-100 in PBS with 1x protease and phosphatase inhibitors. Phagosomes were isolated as described (8) using latex beads (Sigma). Briefly, WT macrophages were incubated with beads diluted 1:50 in 10% FBS/DMEM. After washing away non-internalized beads with PBS, the cells were scraped into 0.1% NP-40 lysis buffer (150 mM NaCl, 20 mM Tris–HCl, 10 mM EDTA, 1.5 mM EGTA, 50 mM NaF, 10 mM $Na_4P_2O_7$, 1 mM Na_3VO_4) containing protease and phosphatase inhibitors, and homogenized by vigorously ejecting the cell suspension through a 22G needle. Cell extracts were centrifuged at 1000×*g* for 10 min at 4°C. The phagosomes were then isolated from the supernatant by sucrose density gradient ultracentrifugation. Phagosomes were solubilized in cold 1% NP-40 lysis buffer.

RNA Isolation and Real-Time RT-PCR: Total RNA was purified using Aurum Total RNA kit (Bio-Rad). Complementary DNA was synthesized with iScript reverse transcriptase (Bio-Rad). Relative quantitation of target mRNA was performed using specific primers (Supplemental Table), SYBR probe (Bio-Rad), and iTaqDNA polymerase (Bio-Rad) on IQ5 thermocylcer (Bio-Rad) and normalized to β-actin.

Statistics Analysis: Data are presented as mean ± SEM. Differences between mean values were determined by one-way ANOVA (Bonferroni's post test), Krushal-Wallis test (Bunn's multiple comparison), Mann-Whitney test, and Student's *t*-test using GraphPad PRISM. Prior to using one-way

ANOVA (Bonferroni's post test) to test for significance, the normality of the sample populations was tested by the Kolmogorov-Smirnov test.

Supplemental References

1. Linton, M. F., J. B. Atkinson, and S. Fazio. 1995. Prevention of atherosclerosis in apolipoprotein E-deficient mice by bone marrow transplantation. *Science.* **267**: 1034-1037.
2. Zhang, W., P. G. Yancey, Y. R. Su, V. R. Babaev, Y. Zhang, S. Fazio, and M. F. Linton. 2003. Inactivation of macrophage scavenger receptor class B type I promotes atherosclerotic lesion development in apolipoprotein E-deficient mice. *Circulation.* **108**: 2258-2263.
3. Lowe, S. W., E. M. Schmitt, S. W. Smith, B. A. Osborne, and T. Jacks. 1993. p53 is required for radiation-induced apoptosis in mouse thymocytes. *Nature.* **362**: 847-849.
4. Li, S., Y. Sun, C. P. Liang, E. B. Thorp, S. Han, A. W. Jehle, V. Saraswathi, B. Pridgen, J. E. Kanter, R. Li, C. L. Welch, A. H. Hasty, K. E. Bornfeldt, J. L. Breslow, I. Tabas, and A. R. Tall. 2009. Defective phagocytosis of apoptotic cells by macrophages in atherosclerotic lesions of ob/ob mice and reversal by a fish oil diet. *Circ Res.* **105**: 1072-1082.
5. Yancey, P. G., J. Blakemore, L. Ding, D. Fan, C. D. Overton, Y. Zhang, M. F. Linton, and S. Fazio. Macrophage LRP-1 controls plaque cellularity by regulating efferocytosis and Akt activation. *Arterioscler Thromb Vasc Biol.* **30**: 787-795.
6. Seimon, T. A., Y. Wang, S. Han, T. Senokuchi, D. M. Schrijvers, G. Kuriakose, A. R. Tall, and I. A. Tabas. 2009. Macrophage deficiency of p38alpha MAPK promotes apoptosis and plaque necrosis in advanced atherosclerotic lesions in mice. *J Clin Invest.* **119**: 886-898.
7. Dowler, S., G. Kular, and D. R. Alessi. 2002. Protein lipid overlay assay. *Sci STKE.* **2002**: pl6.
8. Vinet, A. F., and A. Descoteaux. 2009. Large scale phagosome preparation. *Methods Mol Biol.* **531**: 329-346.
